# Self‐Organization of Redeposited Binder Nanoparticles in Cubic Boron Nitride Materials

**DOI:** 10.1002/smll.202510519

**Published:** 2025-11-20

**Authors:** Manuela Pacella, Stuart Robertson, Jialin Dong, Amir Badiee

**Affiliations:** ^1^ Wolfson School of Mechanical Electrical and Manufacturing Engineering Loughborough University Loughborough Leicestershire LE11 3TU UK; ^2^ Loughborough Materials Characterisation Centre LMCC Loughborough University Loughborough Leicestershire LE11 3TU UK; ^3^ School of Engineering and Physical Science University of Lincoln Lincoln LN6 7TS UK

**Keywords:** coatings, laser surface engineering, polycrystalline cubic boron nitride, titanium diboride, titanium nitride

## Abstract

Herein, the previously unrealized ability to grow microspherical agglomerates using nanosecond laser irradiation is demonstrated. In as short as 1 min, microagglomerates with nanoparticles of Titanium (both spherical and woven‐like) are grown in air. For the first time, it is demonstrated that surface nano‐structuring of cubic boron nitride composites is possible by self‐organization of redeposited Ti nanoparticles, at various energy levels and in the vaporization regime of laser‐matter interaction. The effect of low‐energy laser processing on the surface integrity (i.e., crystallization, morphology) and chemical transformations of polycrystalline cubic boron nitride composites is investigated, with particular emphasis on understanding the effect of laser fluence on chemical transformation at the interface between hard phase and binder. The structure of the newly formed TiB_2_ and TiN is investigated through Scanning Electron Microscopy (SEM), Energy Dispersive X‐Ray Spectroscopy (EDS), Focus Ion Beam Milling (FIB), High Resolution Transmission Electron Microscopy (HRTEM), and X‐Ray diffraction spectroscopy (XRD).

## Graphical abstract

The article reveals a novel approach to forming micro‐spherical agglomerates through nanosecond laser irradiation, enabling rapid growth of titanium nanoparticles in air. It explores the self‐organisation of redeposited Ti nanoparticles on cubic boron nitride composites, detailing structural and chemical transformations that pave the way for innovative TiB_2_ and TiN nano‐coatings, revolutionising cutting tool manufacturing.

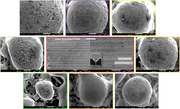

## 1 Introduction

The need for high‐performing ultra‐hard materials used in machining tools is constantly growing, as manufacturers strive to maximize production efficiency and reduce costs,^[^ ^1^ ^]^ as well as having to adapt the synthesis and manufacturing processes to comply with increasingly more stringent environmental legislations. Boron nitride has a crystalline structure, like that of carbon. The tetrahedral arrangement of boron atom surrounded by four nitrogen atoms with sp3 hybridization and very strong ionic‐covalent bonding is what convey PcBN its excellent mechanical properties (Knoop Hardness 43–46 GPa, fracture toughness 5 MPa m^0.5^).^[^ ^2^ ^]^ The nature of this bonding and structure leads to a short bond length and, subsequently, a higher value of bulk modulus. These structural properties make PcBN a material with excellent abrasion resistance, excellent thermal stability (thermal conductivity at 20 °C W m^−1^ K^−1^), and good oxidation‐resistance properties, making it the preferred choice for machining of ferrous metals and related alloys.^[^ ^2^ ^]^ The development of the ideal cutting tool material is an ongoing aim for materials manufacturers of the 21st century, coupled with increased demand of materials’ customization from cutting tool manufacturers and end‐users. Current industrial standards define the ideal cutting tool material having: i) a high hardness (to be retained at elevated temperatures) ii) a high toughness, to enhance resistance to chip/fracture during cutting operations; and iii) a high wear resistance to enable long tool life.^[^ ^2^, ^3^ ^]^ Coatings are often developed to provide additional customization to the material's properties and extend the tool life in use. However, multi‐layer nano/micro‐coatings require an additional manufacturing step, which contributes to added costs to the final product. Furthermore, when textured cutting tools are used, coating represents a huge challenge for manufacturers as this step should be done post‐texturing, where the main issue is achieving a uniform coating across the textured surface of the tool. Laser‐based processes have gained interest in the texturing of difficult‐to‐cut (hard and ultra‐hard) composites. State of the art research investigated pulsed laser ablation as a technique to manufacture freeform textures on PcBN from micro^[^ ^3^, ^4^, ^5^, ^6^, ^7^ ^]^ to macro scale.^[^ ^3^, ^8^, ^9^ ^]^ The effect of different rastering paths on the ablation depth of high‐cBN content composites has been investigated.^[^ ^10^ ^]^ It was found that at lower pulse energies, a higher deepening rate in shallow kerfs (ablated grooves) can be achieved; and that higher pulse energies facilitate a higher deepening rate in deeper grooves and higher maximum cutting depths. The effect of recoil pressure on the resulting surface during ablation of ultra‐hard composites was also studied. Hoffman investigated the ablation of polycrystalline graphite using a 355 nm wavelength laser (pulse energy 1–120 J cm^−2^) reporting that the ablation mechanism in polycrystalline graphite is a result of recoil pressure, not just the processes of evaporation and explosive boiling. Similar observations have been made in PCD and PcBN materials.^[^ ^11^, ^12^, ^13^, ^14^ ^]^ Laser processing and its effect on the topography and hardness of both PCD and PcBN have also been examined.^[^ ^15^, ^16^, ^17^, ^18^ ^]^ Using a laser fluence below the ablation threshold of the composite materials, the hardness of the treated material can be increased for higher feed speed conditions to a certain depth in the material. Grove et al.^[^ ^15^ ^]^ found that for lower feed speeds, the ablation of a medium grade PcBN composite (≈65%) becomes more pronounced, greatly increasing the surface roughness of the processed area. A previous study also employed femtosecond laser processing in a solvent to create coatings in TiN, TiON,TiC/TiOC, and SiC. Highly stable flat surfaces were achieved. Using a low repetition rate aids the formation of microconical and porous structures.^[^ ^19^ ^]^ A further study^[^ ^20^ ^]^ reported on the synthesis of Titanium diboride (TiB_2_) nanoparticles within polycrystalline boron nitrides (cBN) through in situ reactions, using high‐pressure, high‐temperature (HPHT) sintering processes. This process typically involves the use of titanium‐coated cBN powders, where the titanium coating reacts with the cBN matrix. These reactions lead to the formation of TiB_2_, along with other titanium‐based compounds such as titanium nitride (TiN). The resulting microstructure of the composite can be tailored by adjusting synthesis parameters, such as temperature and pressure, allowing for controlled morphology of the TiB_2_ nanoparticles. The in situ formation of TiB_2_ within cBN primarily occurs during HPHT sintering, where titanium‐coated cBN powders are subjected to extreme conditions.^[^ ^20^ ^]^ The titanium from the coating chemically interacts with the boron in cBN to form TiB_2_. Due to titanium's high reactivity, especially at elevated temperatures, other compounds such as TiN may also form during the reaction. This synthesis process is often exothermic, generating localized heat that can promote further reactions and affect the final microstructure. Various synthesis methods exist for producing TiB_2_, each with unique advantages. High pressure high temperature (HPHT) sintering remains the most common, where Ti‐coated cBN powders are processed under extreme conditions to induce the desired reactions.^[^ ^20^ ^]^ Alternatively, mechanical alloying, where titanium and boron powders are milled together under high energy, can also produce TiB_2_. Plasma synthesis methods, which involve vapor‐phase reactions between titanium and boron in a plasma flow, offer another route to form TiB_2_ nanoparticles. Control over the morphology and distribution of TiB_2_ is achievable by modifying synthesis parameters, especially temperature. At lower temperatures, TiB_2_ tends to form whisker‐like structures, while higher temperatures favor the development of plate‐like or granular forms. The particle size and dispersion of TiB_2_ nanoparticles significantly influence the mechanical properties of the resulting cBN composite, including its hardness and wear resistance.^[^ ^21^, ^22^ ^]^ These TiB_2_‐reinforced cBN composites are particularly valued in industrial applications such as cutting tools and abrasives due to their enhanced hardness and durability. In addition to improving mechanical properties, the inclusion of TiB_2_ can also affect the composite's electrical and thermal behavior, broadening its application range. Related materials often accompany TiB_2_ in these composites. Titanium nitride (TiN) is a frequent byproduct due to titanium's reactivity with nitrogen present in the boron nitride matrix. Additionally, alloying elements such as aluminum can be introduced to influence reaction kinetics and phase stability, offering further tunability in the composite's final properties. Previous research^[^ ^21^ ^]^ investigated the in situ synthesis of titanium diboride (TiB_2_) with varying morphologies in polycrystalline cubic boron nitride (PcBN) composites, achieved under different sintering temperatures. Utilizing a cBN–Ti–Al system subjected to ultra‐high pressure (≈5.5 GPa), they demonstrated that TiB_2_ morphology is highly sensitive to sintering temperature. At relatively low temperatures (≈1100 °C), TiB_2_ formed as whisker‐like structures. As the temperature increased, its morphology transitioned to plate‐like and rod‐shaped forms, eventually evolving into granular particles at approximately 1600 °C.^[^ ^21^ ^]^ These results underscored the pivotal role of sintering temperature in dictating TiB_2_ morphology, offering a pathway for designing PcBN composites with tailored mechanical properties. Rao et al.^[^ ^23^ ^]^ investigated the development and performance of multilayer coatings composed of titanium diboride (TiB_2_) and carbon, with the goal of enhancing the mechanical and tribological properties of TiB_2_. While TiB_2_ is known for its high hardness and wear resistance, its brittleness limits practical applications. To overcome this, the researchers designed multilayer stacks by alternating TiB_2_ with thin carbon layers, aiming to combine the load‐bearing capacity of the ceramic with the lubricity and crack‐arresting ability of carbon. The coatings were fabricated using DC magnetron sputtering with substrate bias onto tool‐steel substrates, and the volume fraction of TiB_2_ was varied between 50% and 95%. This study demonstrated that TiB_2_ coatings offer a promising solution for applications requiring wear‐resistant surfaces with both mechanical durability and enhanced lubricity, such as in tooling and cutting applications. Furthermore, tuning the layer composition and structure allows for the design of advanced coatings that overcome the limitations of monolithic ceramics. Another study^[^ ^24^ ^]^ reported that laser‐based additive manufacturing can be successfully used on Ti6Al4V alloy powder mixed with varying types of BN powder (including cubic BN) to allow simultaneous formation of TiB and TiN phases in situ. High temperatures from the laser interaction promote reactions between titanium and BN, producing TiN dendrites distributed within a Ti matrix. The TiN content increased with higher BN loading, which enhanced stiffness and wear resistance significantly.^[^ ^24^ ^]^ Surface nitriding performed with a laser beam in a nitrogen‐rich atmosphere also led to the formation of graded coatings composed of TiN and Ti_2_N dendrites within an α‑Ti matrix. Higher laser power and multiple scans amplify the TiN‐rich dendritic structure, resulting in notably improved hardness and wear resistance.^[^ ^25^ ^]^ Laser power, number of passes, nitrogen partial pressure, and BN concentration all influence the TiN morphology, higher energy input generally yields coarser, better‐developed TiN dendrites and increased TiN fraction within the coating.^[^ ^25^ ^]^ Wen et al., in their review paper^[^ ^26^ ^]^ reported how in situ TiN and TiB reinforcements are generated during laser cladding or deposition on titanium‐based alloys. Mechanisms such as Marangoni convection in the molten pool, rapid solidification, and nitrogen gas diffusion lead to cellular or dendritic TiN morphologies. TiN particles also act as nucleation sites that refine the microstructure and enhance mechanical strengthening.^[^ ^26^ ^]^ While most studies focus on Ti–BN systems in titanium alloys, the same chemical principles apply when using PcBN or Ti‐coated cBN in laser processing. High local temperatures provided by the laser can induce TiN formation where Ti from the coating or matrix reacts with nitrogen from cBN, producing TiN‐rich dendrites, coatings, or interfacial reaction layers. These structures can significantly affect hardness, wear resistance, and bonding characteristics. Currently, there is no documented research that explicitly investigates the laser‐induced formation of titanium nitride (TiN) and titanium diboride (TiB_2_) within PcBN composites. However, analogous studies on laser nitriding of titanium alloys demonstrate that, under nitrogen‐rich environments, titanium readily reacts to form TiN dendrites. These findings suggest that similar mechanisms may be applicable to PcBN systems containing metallic titanium phases, such as in the binder material, where laser irradiation in a nitrogen atmosphere could theoretically promote in situ TiN formation through comparable nitridation reactions. While wear performance and behavior of PcBN tools are somewhat documented for varying grades of cBN content and binder compositions, extensive research continues on the properties of PcBN composites, including investigations into different binder materials and the effect of sintering conditions. The response of PcBN to new low‐energy processes has not been reported, and numerous factors must be considered regarding their influence on laser‐material interactions. Since different grades of PcBN serve various machining applications, improved designs for novel grades with functionalized properties are constantly proposed by materials manufacturers, while new geometries are designed by cutting tool manufacturers. New materials design mostly focuses on the crystal/binder architectures while using conventional powder preparation techniques and sintering methods (i.e., High Pressure High Temperature). However, currently no technology focuses on a combined approach of designing both mechanical properties and cutting tool geometry concurrently. The challenging industrial aspect of machinability optimization necessitates simultaneous consideration of tool performance, cutting conditions, and wear patterns to achieve the ideal cutting process. For instance, machining a metallic material with high adhesion and ductility requires selecting a tougher tool, applying a coating, and implementing micro‐geometry while machining at an increased cutting speed. In this context, the newly proposed process aims to control the manufacture of hierarchical structures in ultra‐hard ceramics for cutting tools, simultaneously enhancing mechanical properties and micro‐nano geometries. It is important to note that the synthesis conditions employed in previous research^[^ ^21^ ^]^ required ultra‐high pressures and controlled environments, limiting practical scalability. In contrast, the present study aims to demonstrate the formation of TiB_2_ under ambient pressure and in air, without the need for specialized sintering conditions. This represents a significant advancement, as it offers a simplified and more accessible route to in situ TiB_2_ synthesis, potentially broadening the applicability of such materials in industrial settings.

*Novelty statement*: While previous studies^[^ ^24^, ^25^, ^26^ ^]^ have demonstrated the in situ formation of TiB/TiN phases during laser processing of titanium alloys, the present work introduces a fundamentally distinct approach in both material system and reaction mechanism. Specifically, this study investigates the in situ formation of TiB_2_/TiN within a PcBN (polycrystalline cubic boron nitride) matrix, a material system not previously explored for this purpose. Moreover, unlike prior work where phase formation primarily occurs through in‐pool reactions in molten Ti alloys, this research reveals a self‐organized re‐deposition mechanism, driven by surface reactions and diffusion during laser interaction. Critically, the process is achieved in ambient air, eliminating the need for inert gas shielding, representing a significant advancement in processing simplicity and environmental adaptability. This combination of a novel material system, distinct phase formation pathway, and air‐processability marks a key breakthrough over prior laser‐based TiB_2_/TiN synthesis methods.

In this context, the aims of this paper are:

- To introduce a novel manufacturing method to grow spherical and woven‐like TiN and TiB2 micro and nano particles from a solid PcBN substrate;
- To understand how laser ablation can be used for the first time to customise materials’ chemistry and the tool's geometry. This is particularly useful for cutting tools where the design of materials/coatings composition is approached separately from the design of the tool's geometry (i.e., chip breaker).

## 2 Results and Discussion

### 2.1 Assessment of the Topographical and Morphological Changes Post Laser Processing

**Figure 1:**
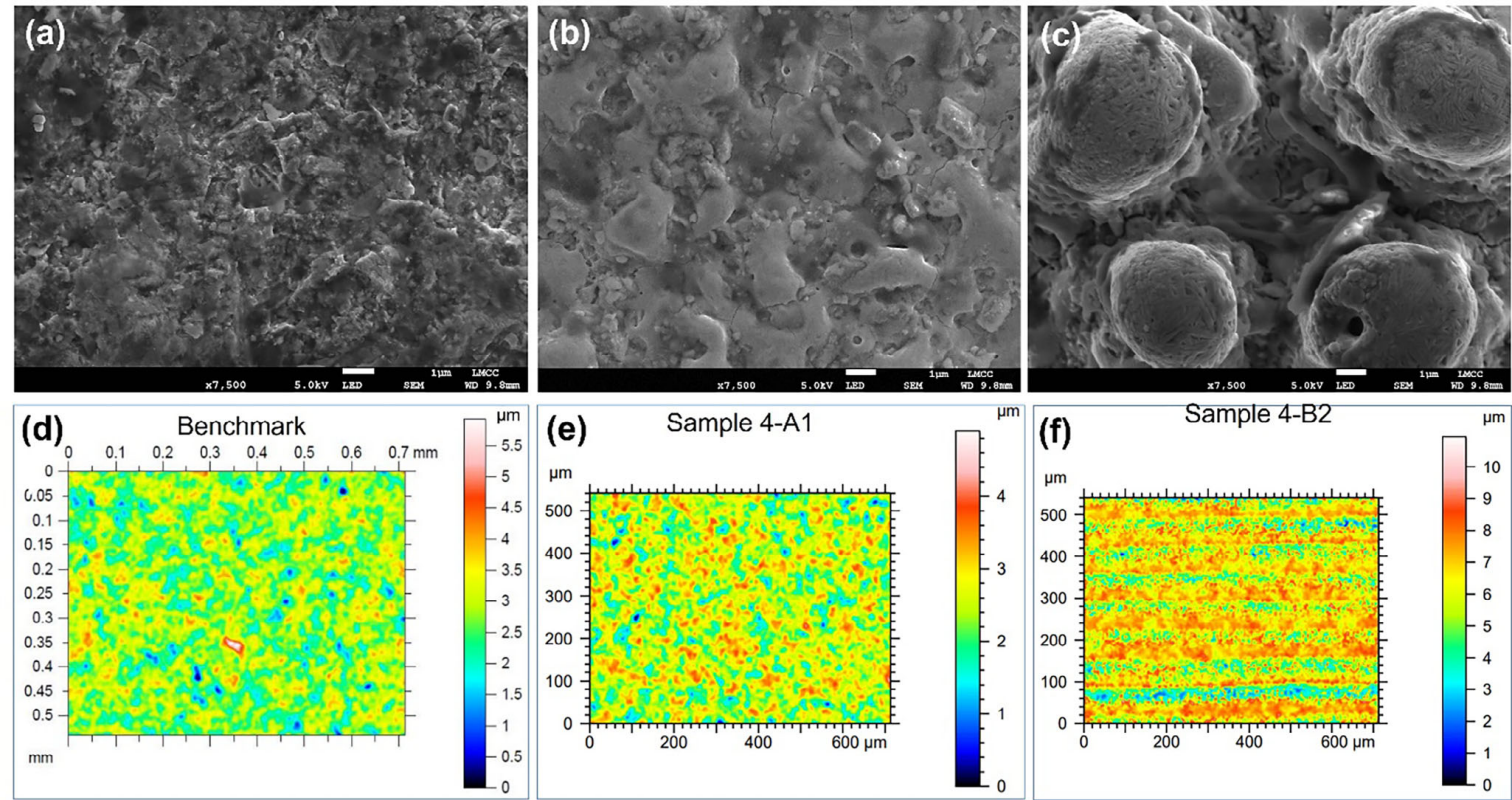
Scanning Electron Microscopy (SEM) images and 3D topographical profile of: a) and d) benchmark Sa = 0.317 µm b) and e) sample 4‐A1 Sa = 0.400 µm c) and f) sample 4‐b2 Sa = 1.010 µm.

Seventeen sets of experiments served as a platform to identify optimal laser parameters in the control of surface roughness and morphology. Plots of the 3D surface roughness Sa for benchmark and processed samples 4A1 and 4B2 are reported in **Figure** **1**. A strong variability of surface roughness was purposely designed and achieved to evaluate the effect this could have on crystallization. All post‐processed samples showed an increase in 3D surface roughness Sa from the benchmark value of 0.317 µm to (for example) 0.400 µm for sample 4‐A1 and to 1.010 µm for sample 4‐B2.

**Figure 2:**
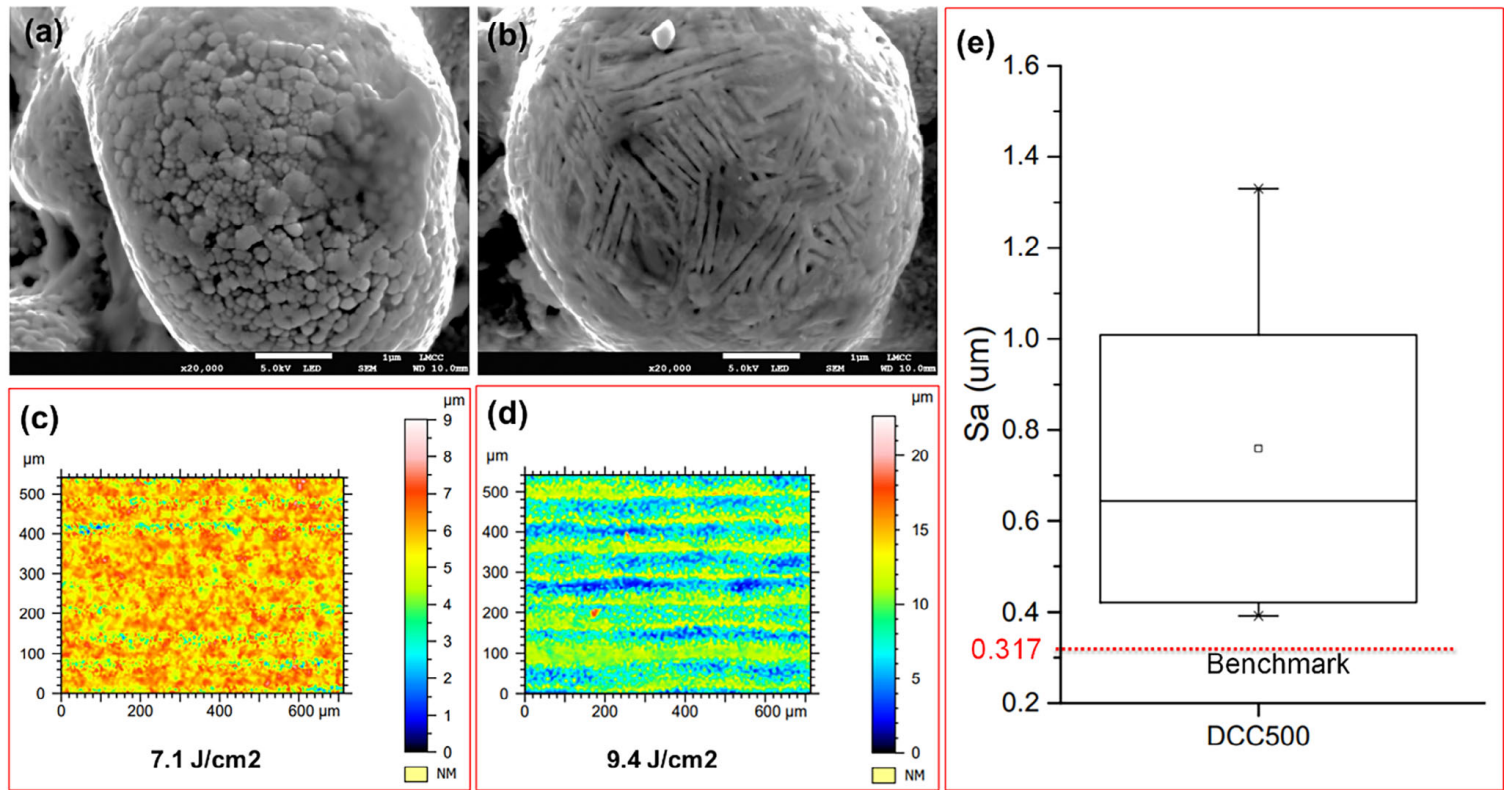
Scanning Electron Microscopy (SEM) images of PcBN laser processed samples: a) condition 8a b) condition 8c, c) 3D topographical profile of condition 8a, Sa = 0.565 µm d) 3D topographical profile of condition 8c, Sa = 2.16 µm; e) boxplot of the 3D roughness Sa measurements of all the laser conditions reported in Table 4.

Two types of growth formation were identified from the seventeen sets of experiments (**Table** **1**), these were named as “spherical” (**Figure** **2a**), and “woven‐like” (Figure 2b). The predominant factor affecting the formation of spherical and woven‐like growth was frequency, pulse duration, and focal distance. To promote the formation of woven‐like structures, lower pulse duration (10 ns) and lower feed speed (400 mm s^−1^) were selected. To promote the formation of bubble‐like spherical nano structures, a longer pulse duration was selected (130 ns). Within each of the two groups, it was possible to achieve a higher degree of control of nano‐particle density, dimensions, and structure by varying additional laser parameters such as track distance, repetition frequency, and feed speed (full range of parameters is reported in Table 1).

**Table 1:** Table of atomic % concentration of averaged EDS spectra from each phase identified. Representative areas (1 to 6) are shown in Figure 12 and (A to C) in Figure 13.

EDS point analysis Sample 11	B	C	N	Al	Ti	Fe	Co	W	Total	Reference to figure
Ti‐C‐N		17.99	8.53	1.22	70.87	0.21	0.26	0.92	100	Area 1 in Figure 12 Area A Figure 13
TiB	45.23	4.37	0.82	0.87	47.83	0.14	0.15	0.6	100.01	Area 2 in Figure 12 Area B Figure 13
BN	50.16		46.8	1.4	1.57	0.02	0.02	0.03	100	Area 3 in Figure 12 Area C Figure 13
EDS point analysis Sample 15	B	C	N	Al	Ti	Fe	Co	W	Total	Reference to Figure
TI‐C‐N	0	18.53	7.77	0.94	71.63	0.14	0.14	0.85	100	Area 4 in Figure 12 Area A Figure 13
TiB	52.08	4.8	0.08	0.85	41.3	0.2	0.14	0.55	100	Area 5 in Figure 12 Area B Figure 13
BN	54.2	0	45.11	0.14	0.54	0	0.01	0	100	Area 6 in Figure 12 Area C Figure 13

Scanning electron microscopy images of samples processed at 7.1 and 9.4 J cm^−2^ are depicted in Figure 2a,b, respectively. Overall, 3D surface roughness Sa quadrupled (from 0.56 to 2.16 µm) when increasing laser fluence (Figure 2e). Typically, rastering paths (grooves) can be seen in the metrological map (Figure 2d), while these are not evident in Figure 2c where a more homogeneous texturing in both directions was achieved. Not only is the increased fluence (and therefore energy density) is responsible for a deeper ablation (and texturing), but also the type of nano particle growth varies. Four‐leaves clover‐like deposits are apparent in Figure 2a while woven‐like structures are seen in scanning electron images in Figure 2b.

**Figure 3:**
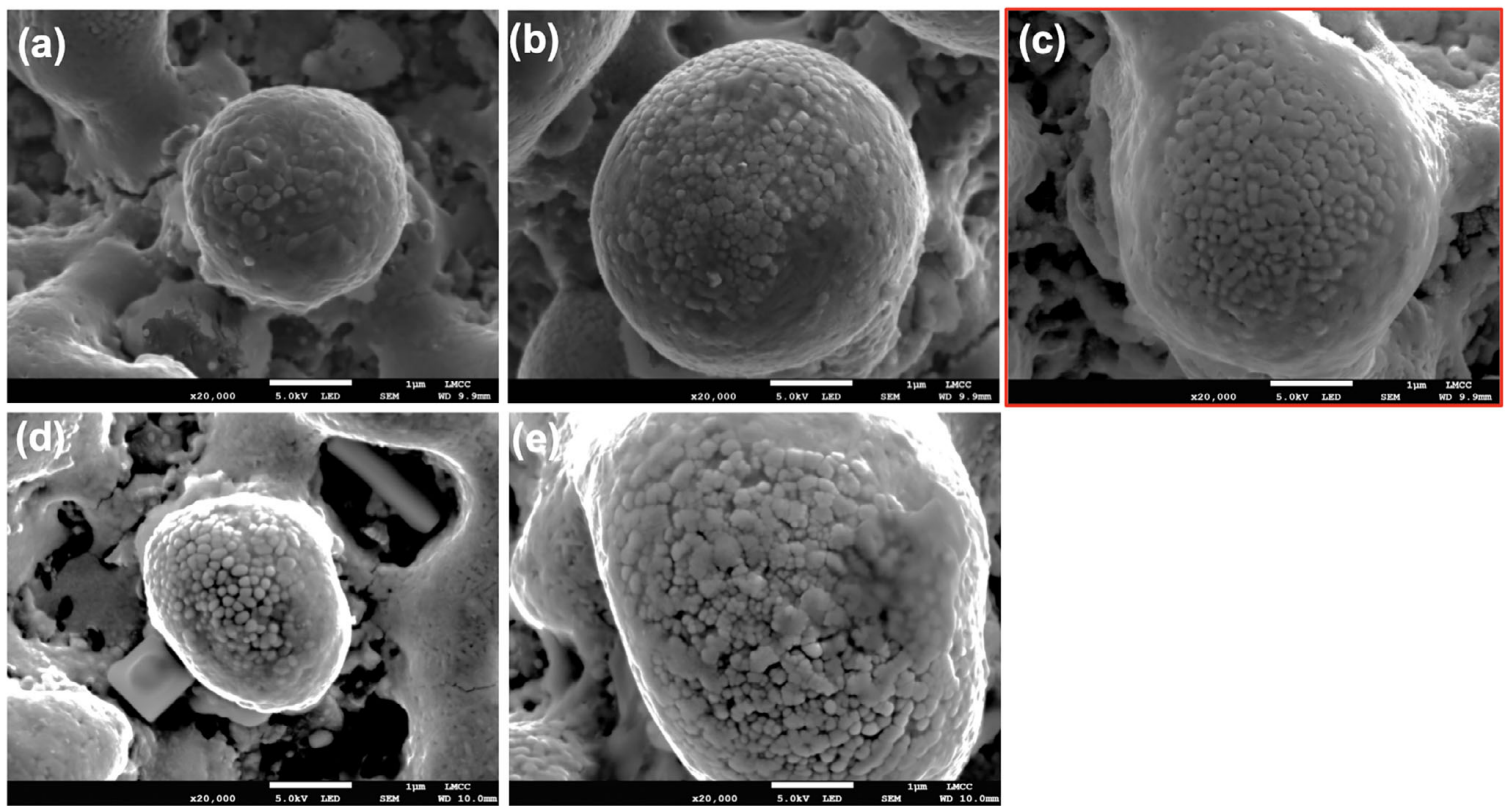
Scanning Electron Microscopy (SEM) images of PcBN laser processed samples: a) condition 15a, b) condition 13b5, c) condition 15c, d) condition 7a, e) condition 8a. Parameters for each of these conditions are reported in Table 4.

**Figure 4:**
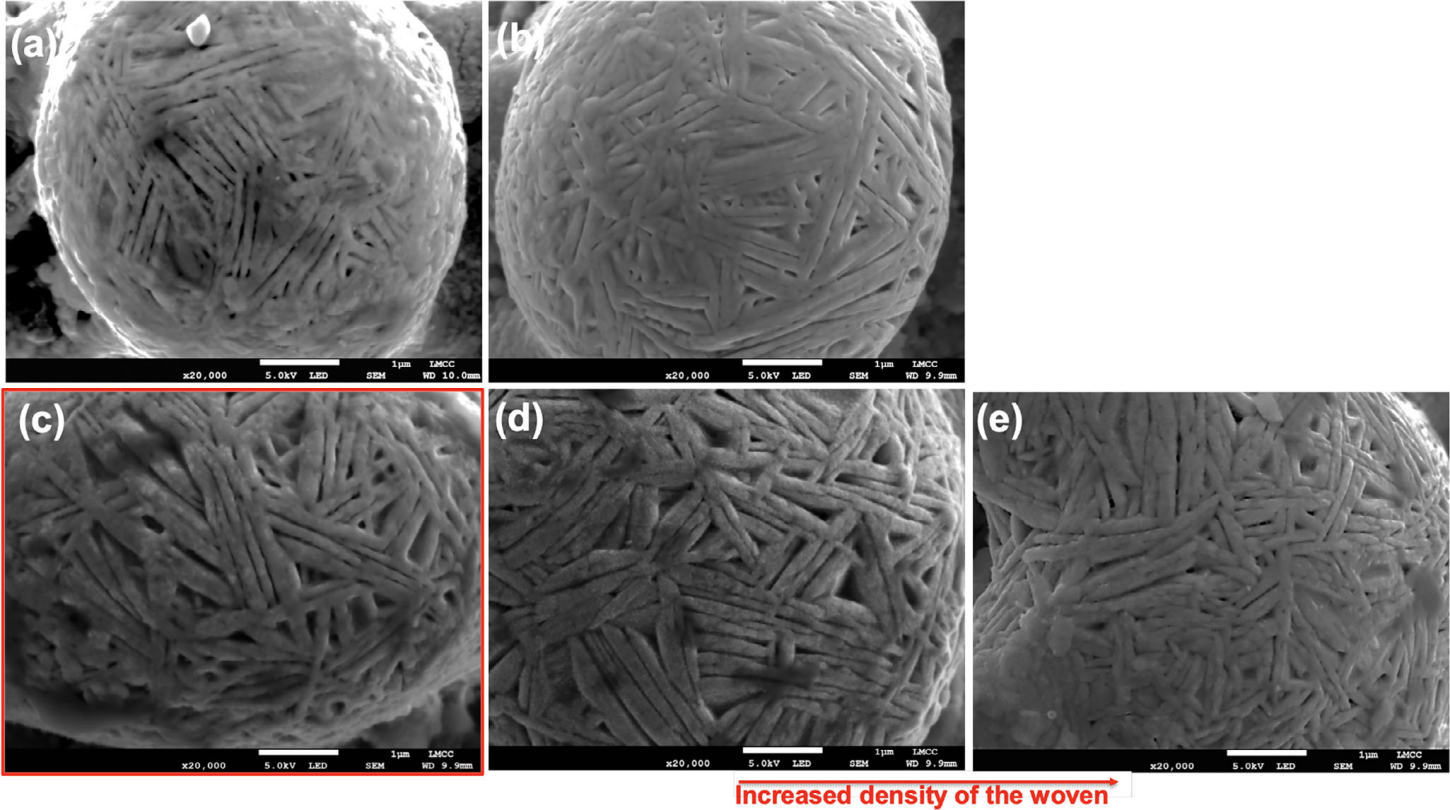
Scanning Electron Microscopy (SEM) images of PcBN laser processed samples: a) condition 8c, b) condition 15b4, c) condition 11c1, d) condition12c1, se) condition 12c2.

The two groups of achieved features are represented in **Figures** **3** and **4**. All the agglomerates are spherical micro‐particles (2‐10 µm range dimension) with distinguished nano particles which are spherical (Figure 3d), four‐leaf clover like (Figure 3e), and woven‐like (Figure 4). It is apparent that spherical particles with round or four‐leaf clover are smaller structures (2–4 µm) than in the case of woven like structures (4–10 µm). These have dimensions ranging from 50 to 200 nm (Figure 3). For the woven‐like structures, the dimensions of the fibres range significantly from 0.5 µm (Figure 4e) to 3 µm (Figure 4c,d). The difference between bubble and woven structures is given by the pulse duration. Woven are produced at 10 ns while bubble at 130 ns. Previous work^[^ ^27^ ^]^ demonstrated that when processing in very short cycles (millisecond pulse duration), the brief cycle leads to localized melting rather than full densification, since viscous flow is limited by the inherently high melt viscosity of metals. At lower energy levels, solid‐state bonding alone is not sufficient, which makes liquid phase sintering or complete melting and solidification essential for achieving effective fusion between particles. The scan rate and laser energy input play a critical role in determining the quality of the sintered parts. However, for scan rate between 50 and 100 mm s^−1^, the sintered density tends to stabilize.^[^ ^27^ ^]^

The achieved bubble shaped textures resemble features which are sometimes achieved via Laser Powder Bed Fusion (LPBF)^[^ ^28^ ^]^ and selective laser melting (SLM) due to balling effects.^[^ ^29^, ^30^ ^]^

Balling in LPBF processes refers to the formation of small spherical droplets or beads during laser processing. However, the term “balling” is used in this paper in a broader morphological sense to describe the formation of spherical features, but the underlying mechanism differs due to the distinct thermal and temporal characteristics of nanosecond laser ablation. In LPBF, balling occurs due to instabilities in a fully developed molten pool, governed by factors such as surface tension gradients (Marangoni flow), melt viscosity, and capillary breakup. This process assumes the powder is completely melted and resolidifies under controlled layer‐by‐layer conditions. However, in the present study, the laser–material interaction regime differs significantly. The nanosecond pulsed fiber laser induces very short heating durations, leading primarily to partial melting, surface evaporation, and re‐deposition of material rather than sustained melt pool formation. Therefore, while spherical particles (“balls”) are observed on the surface, their origin is more likely related to localized vaporization and condensation or melt ejection due to explosive boiling, not the melt instability mechanism characteristic of LPBF. Zhu et al.^[^ ^29^ ^]^ also describe how spherical titanium carbide (TiC) particles form through an in situ reaction during combustion synthesis in a Ni–Ti–C system. The process unfolds as a sequence of thermally driven transformations beginning with intense localized heating. Under these high‐temperature conditions, titanium and carbon react and dissolve into the molten nickel matrix, initiating the early stages of TiC formation. One of the significant challenges in laser processing is melt instability. During laser scanning, molten material tends to form cylindrical tracks, but these are susceptible to capillary (Rayleigh) instabilities. As a result, the molten tracks break up into spherical agglomerates, a phenomenon known as “balling,” which severely reduces part density. The likelihood of this breakup is influenced by factors such as scan speed, melt track diameter, and laser power. Higher scan speeds reduce the size of the melt pool and make it more prone to instability. The formation of perfectly spherical bubbles observed during processing can be attributed to three primary factors: I) High Processing Temperature: the extremely elevated temperatures achieved during laser processing play a critical role. Jin et al. (2009)^[^ ^31^ ^]^ demonstrated that in the high‐temperature synthesis of TiC, increased combustion temperatures reduce the anisotropic growth of TiCx, resulting in more spherical morphologies. This phenomenon is evident at the bottom of the laser tracks, corresponding to regions of highest energy, where characteristic balling occurs alongside morphologically distinct bubbles and woven structures, exhibiting varying strut thicknesses and bubble sizes. II) Surface Energy Minimization: the spherical geometry also results from the system's tendency to minimize surface energy. Since a sphere possesses the lowest surface‐to‐volume ratio among all geometries, it represents the thermodynamically most stable configuration under such constraints. III) Rapid Solidification: the rapid cooling inherent to laser ablation in air inhibits the development of complex, faceted growth modes such as dendritic structures. Consequently, the molten material solidifies quickly into dense, spherical particles. In our results the reactive nature of combustion synthesis itself further reinforces this outcome. Because the reaction is highly exothermic and localized, it creates an environment where nucleation is fast and uniform, favoring the consistent formation of fine, spherical TiN and TiB2 particles. However, we also noted that when cooling occurs more slowly (for example when feed speed is lower or energy density is higher), non‐spherical morphologies such as dendritic structures did not emerge, oppositely to what was previously demonstrated in research on similar compounds (i.e., TiC structures).^[^ ^29^ ^]^

### 2.2 Evaluation of Chemical Changes and Recrystallisation Formation Post Ablation

#### 2.2.1 Characterization via FIB‐HRTEM

To investigate the microstructural and chemical evolution of the laser‐processed PcBN composites, a combination of advanced characterization techniques was employed. Site‐specific cross‐sectional lamellae were prepared using focused ion beam (FIB) milling to facilitate high‐resolution imaging and compositional analysis of reaction zones and interfacial regions. This approach enabled precise targeting of areas influenced by laser‐induced thermal gradients and allowed for detailed examination of microstructural transformations at the sub‐micron scale.

**Figure 5:**
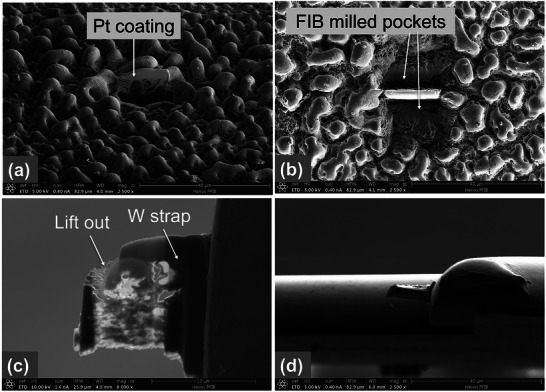
SEM images of the FIB procedure for lamella preparation and extraction: a) Pt coating; b) milling and thinning; c) W strapping technique and lift‐out of a lamella; d) lamella mounting onto a support grid.

**Figure 6:**
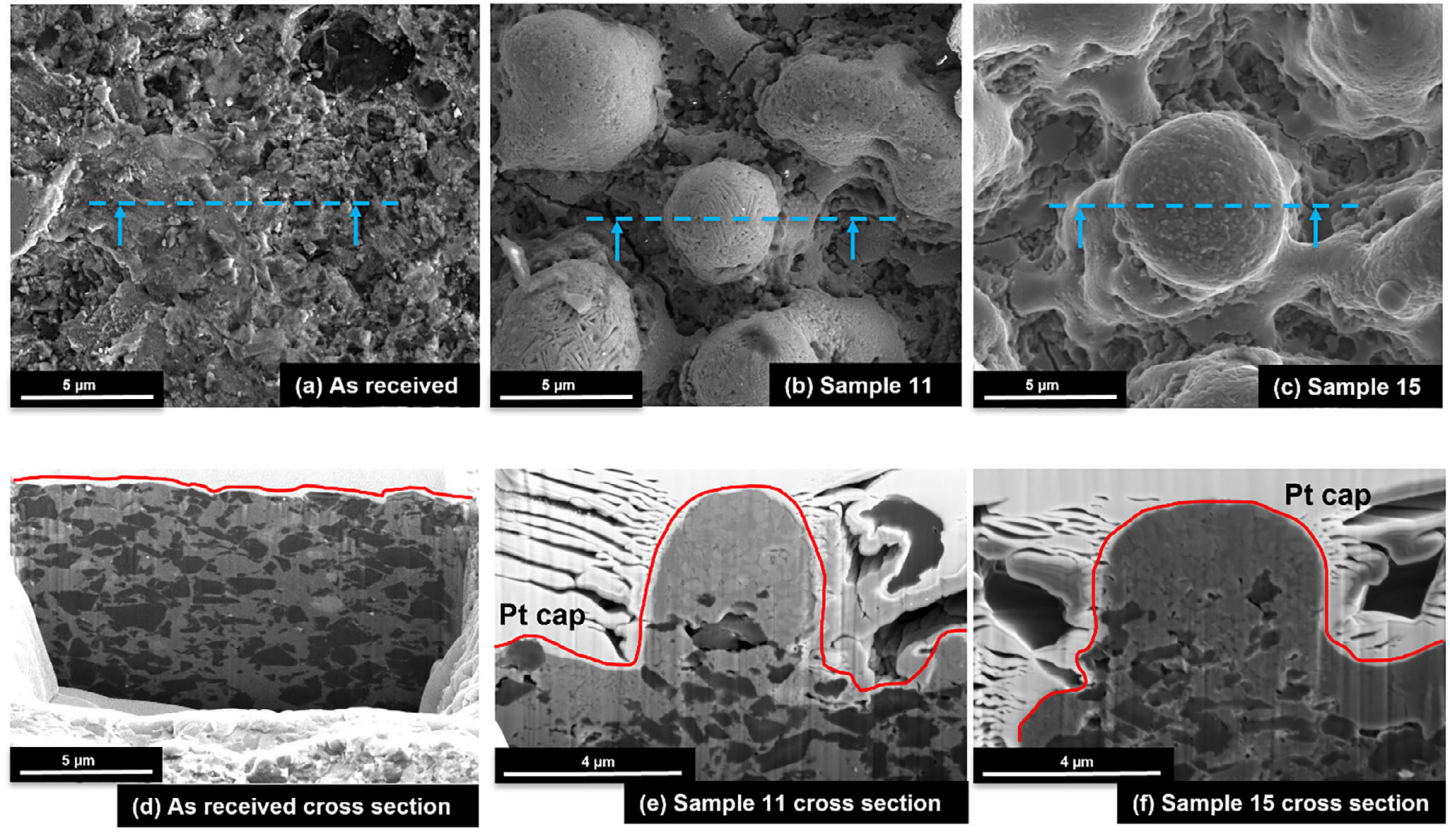
SEM images of (a) as received, b) sample 11 woven structure, and c) sample 15 bubble structure, blue lines denote FIB cross section location. d–f) FIB cross‐section electron images of the as received d), sample 11 e) and sample 15 f). Red lined denote the platinum capping (Pt coating) used for protecting the sample surface.

To preliminarily analyze the chemical composition (qualitatively and quantitatively) of the elements present in the post‐processed sample, Energy Dispersive X‐ray Spectroscopy (EDS) was carried out in mapping mode for four of the laser‐processed samples: two with bubble/spherical nano‐features and two with woven‐like particles. The methodology followed for thew preparation of these samples is reported in **Figure** **5** in which four steps are needed for the lamella preparation and extraction^[^ ^32^ ^]^: a Pt coating, milling and thinning, W strapping technique, and lift‐out of a lamella; finally, lamella mounting onto a support grid for further analysis via High Resolution Transmission Electron Microscopy (HRTEM). Top view of SEM images of some of the analyzed samples is reported in **Figure** **6**, which also includes cross sections of the FIB lamellas prepared for EDS analysis for two of the laser‐processed samples and for the benchmark.

**Figure 7:**
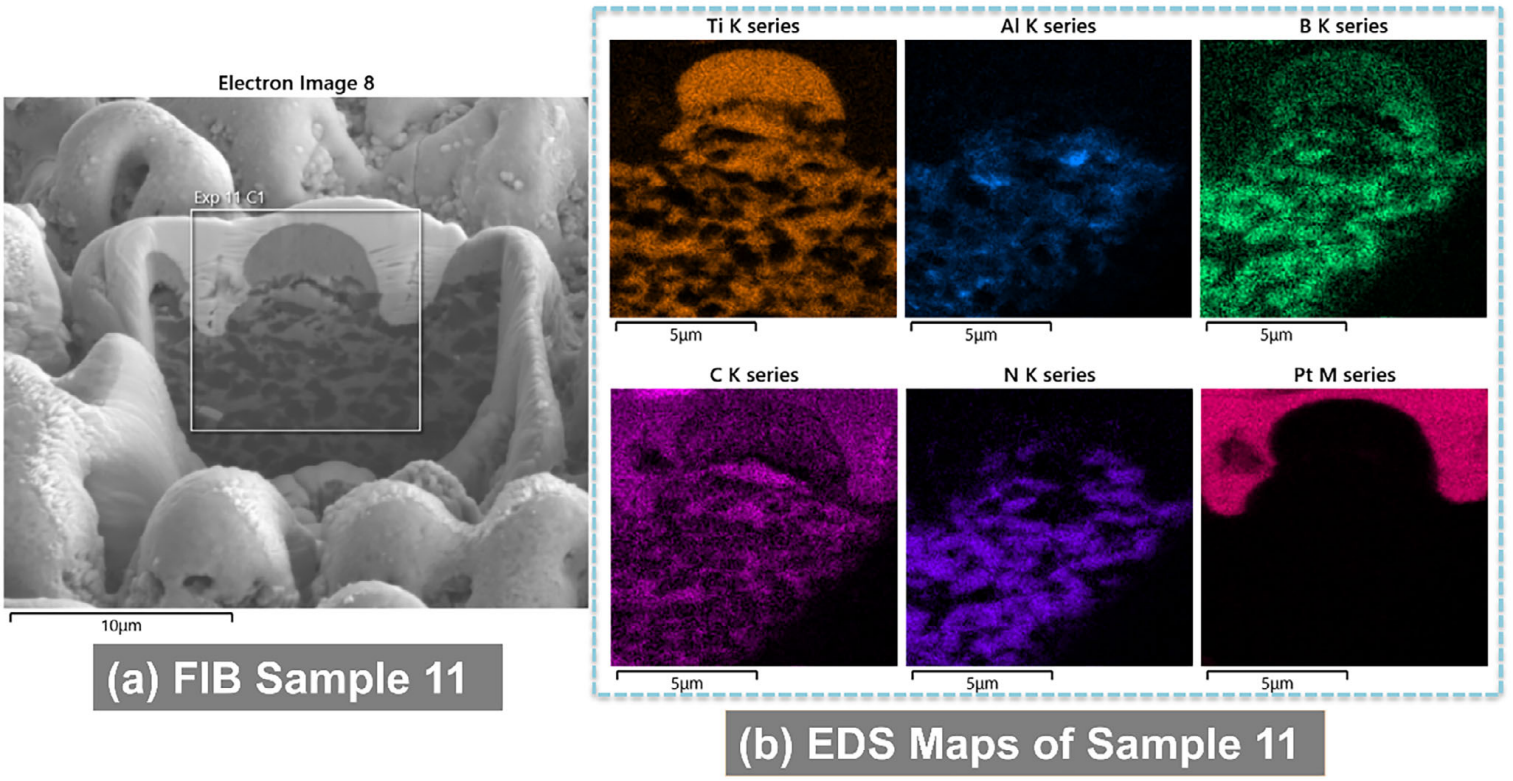
SEM images of the FIB cross section of Sample 11 a) and its respective EDS maps of chemical elements b).

**Figure 8:**
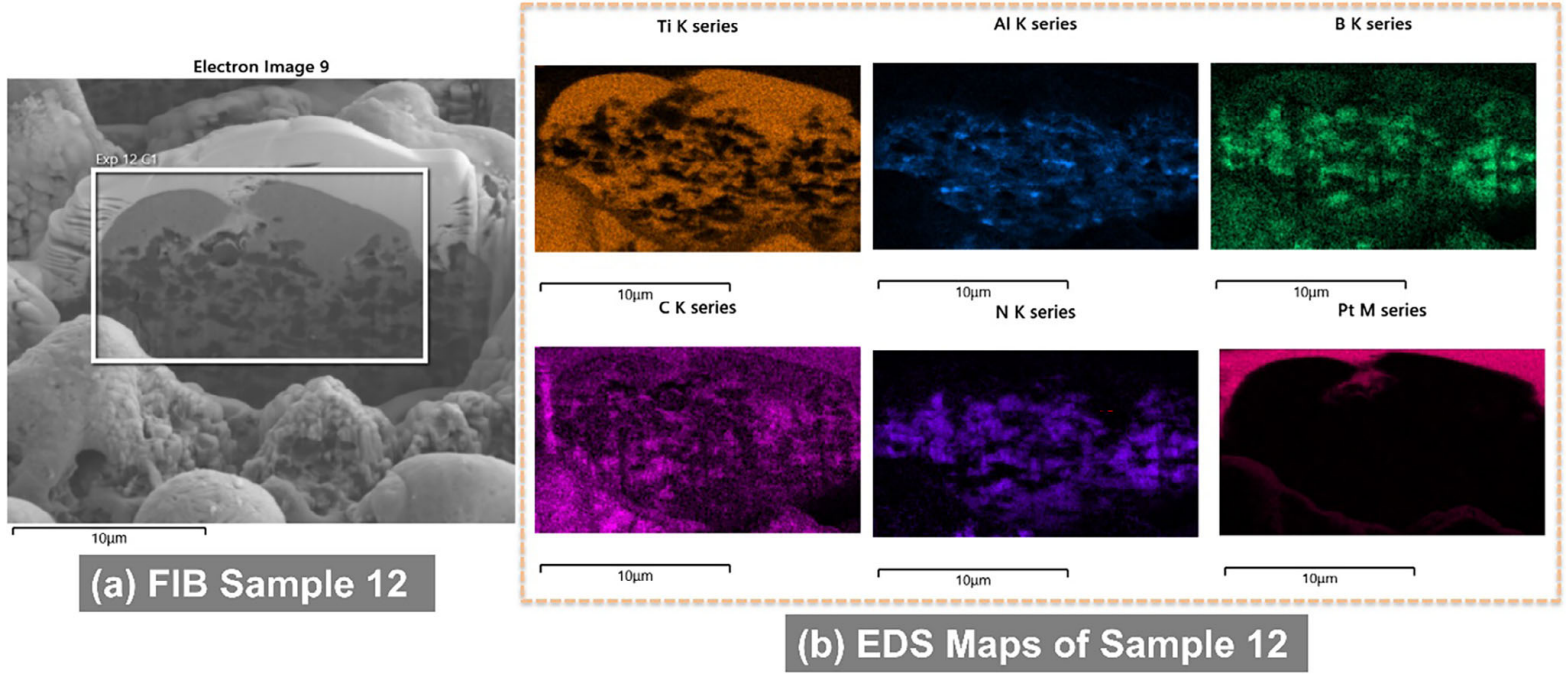
SEM images of the FIB cross section of Sample 12 a) and its respective EDS maps of chemical elements b).

**Figure 9:**
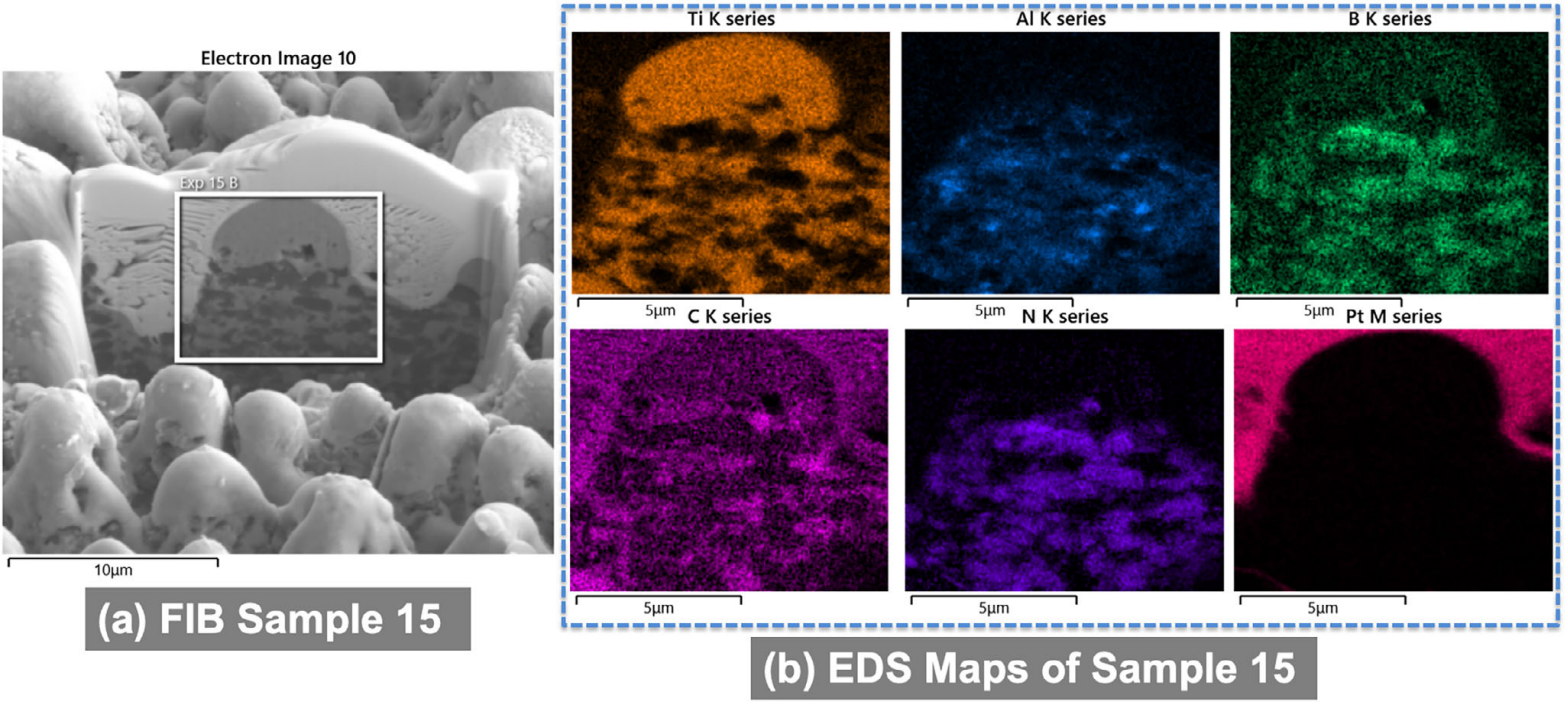
SEM images of the FIB cross section of Sample 15 a) and its respective EDS maps of chemical elements b).

**Figure 10:**
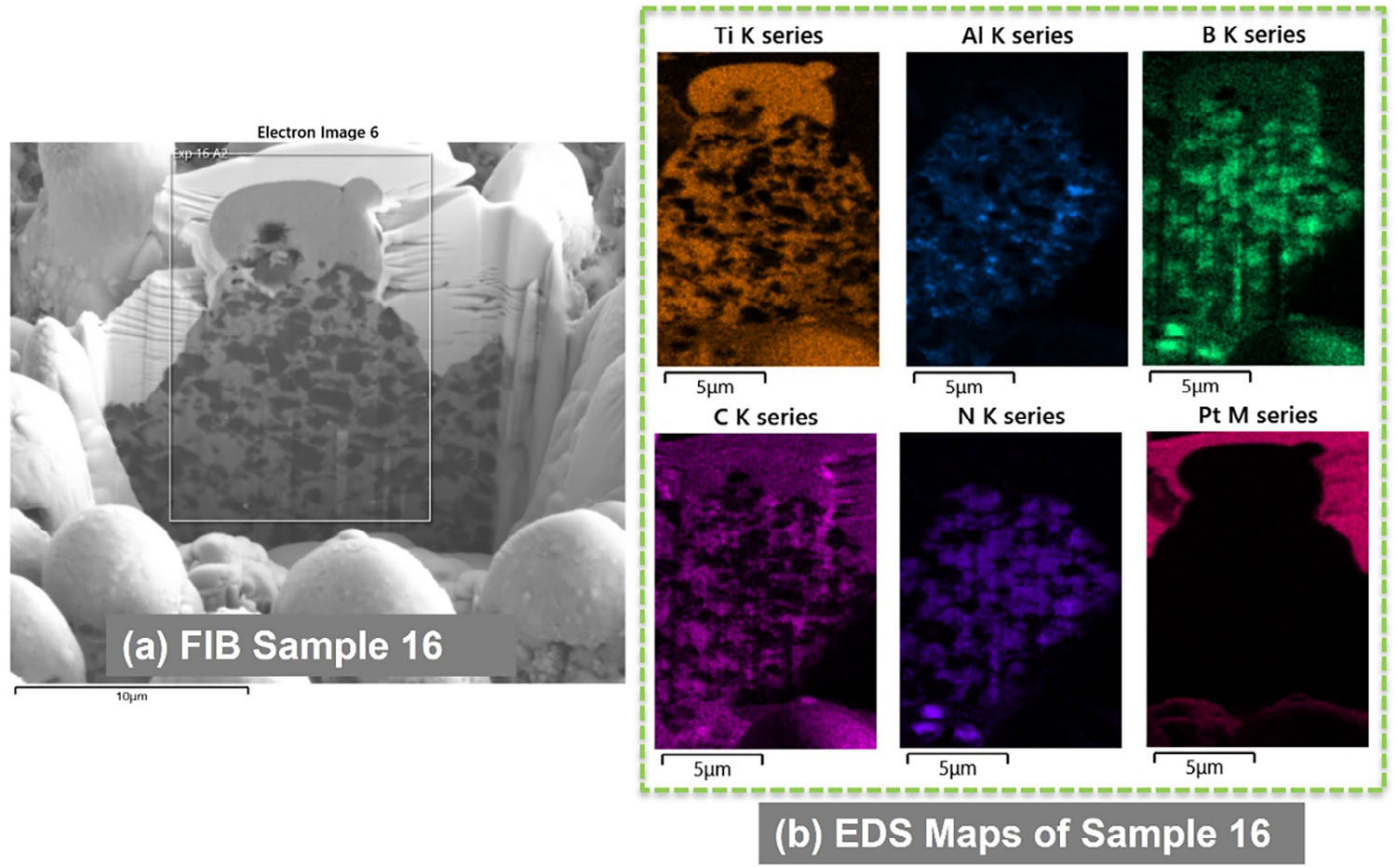
SEM images of the FIB cross‐section of Sample 16 a) and its respective EDS maps of chemical elements b).

EDS analysis revealed localized redistribution of titanium, boron, and carbon. Boron and titanium were enriched in micro‐agglomerates (**Figures** **7**b, **8**b, **9**b, **10**b), suggesting the formation of new compounds during rapid solidification in air. EDS elemental maps showed Ti‐rich regions overlapping with areas of boron accumulation (Figures 7, 8, 9, 10), strongly indicating the in situ formation of boride compounds, such as TiB or TiB_2_, consistent with previous findings in laser‐processed Ti–B systems.^[^ ^33^ ^]^

A major difference between the four samples was not noticed, and therefore, two of the four samples were selected for further analysis involving HRTEM and XRD: Sample 11, which is representative of a woven structure, and Sample 15, which is representative of a bubble structure.

**Figure 11:**
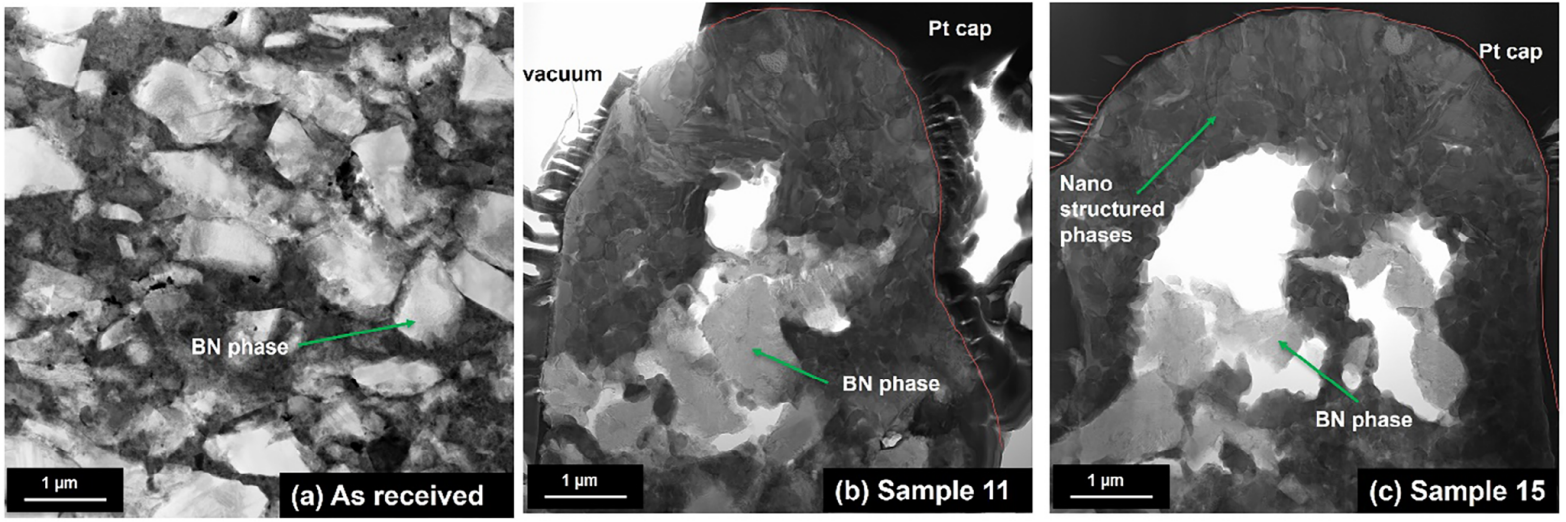
STEM Bright field images of as received sample a), woven‐like features in Sample 11 b), and bubble/spherical features in Sample 15 c).

Site‐specific FIB cross‐section analysis was used to assess the microstructural differences following laser patterning. The as received microstructure (**Figure** **11**a) shows the PcBN matrix with BN inclusions. Following laser processing (Figure 11E,F), the Ti rich phase has been melted (increased in volume), and the BN phase is maintained predominantly in the bulk. Following FIB cross‐section analysis, samples of the as‐received material, samples 11 and 15 were analyzed in further detail using Scanning transmission electron microscopy (STEM) Energy Dispersive X‐ray Spectroscopy (EDX). One Sample was selected with the bubble/spherical nano‐features particles (Figure 11c) and another one with woven‐like particles (Figure 11b).

**Figure 12:**
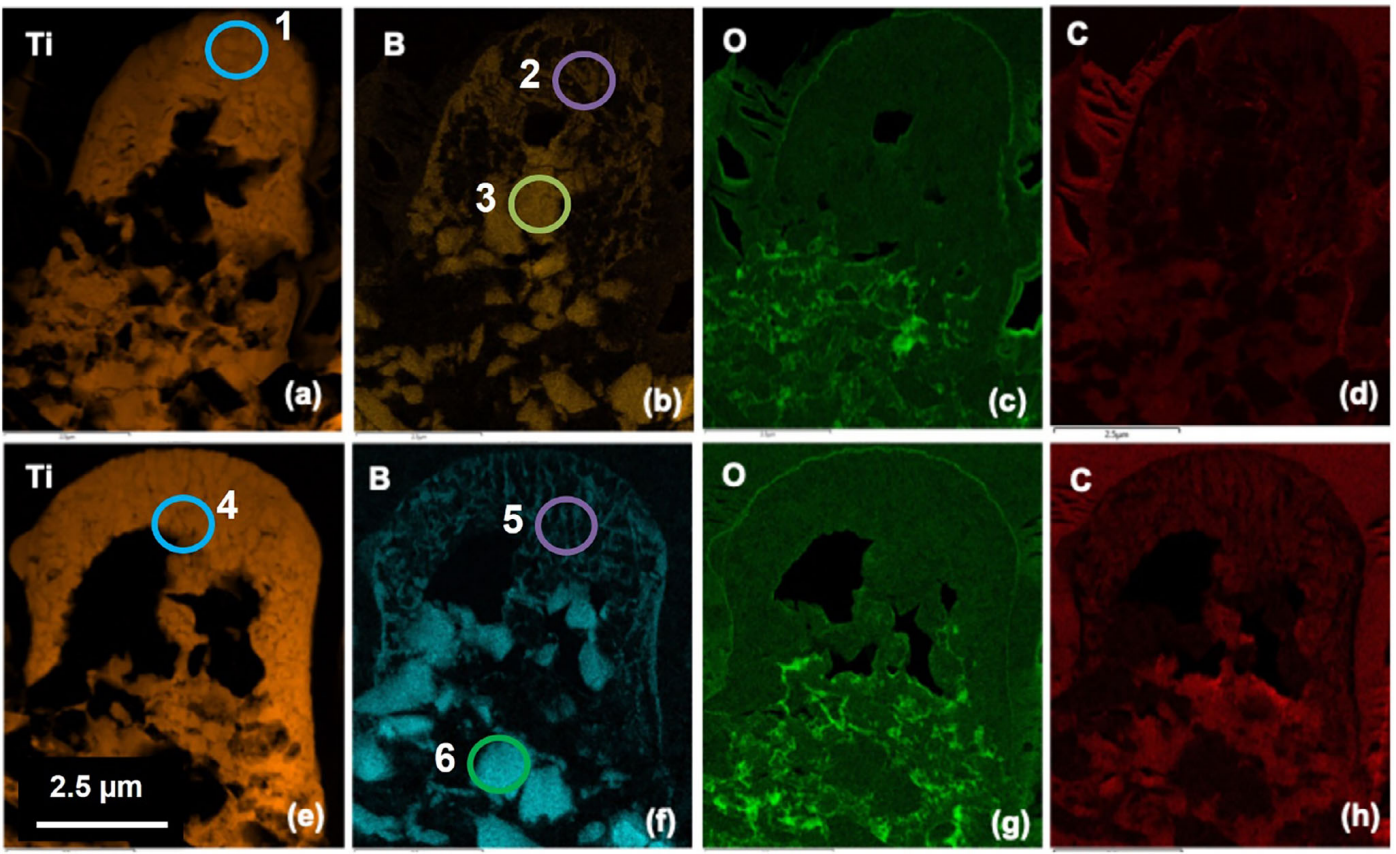
Atomic % STEM EDX Maps (a‐d for sample 15 and e to h for sample 11).

**Figure 13:**
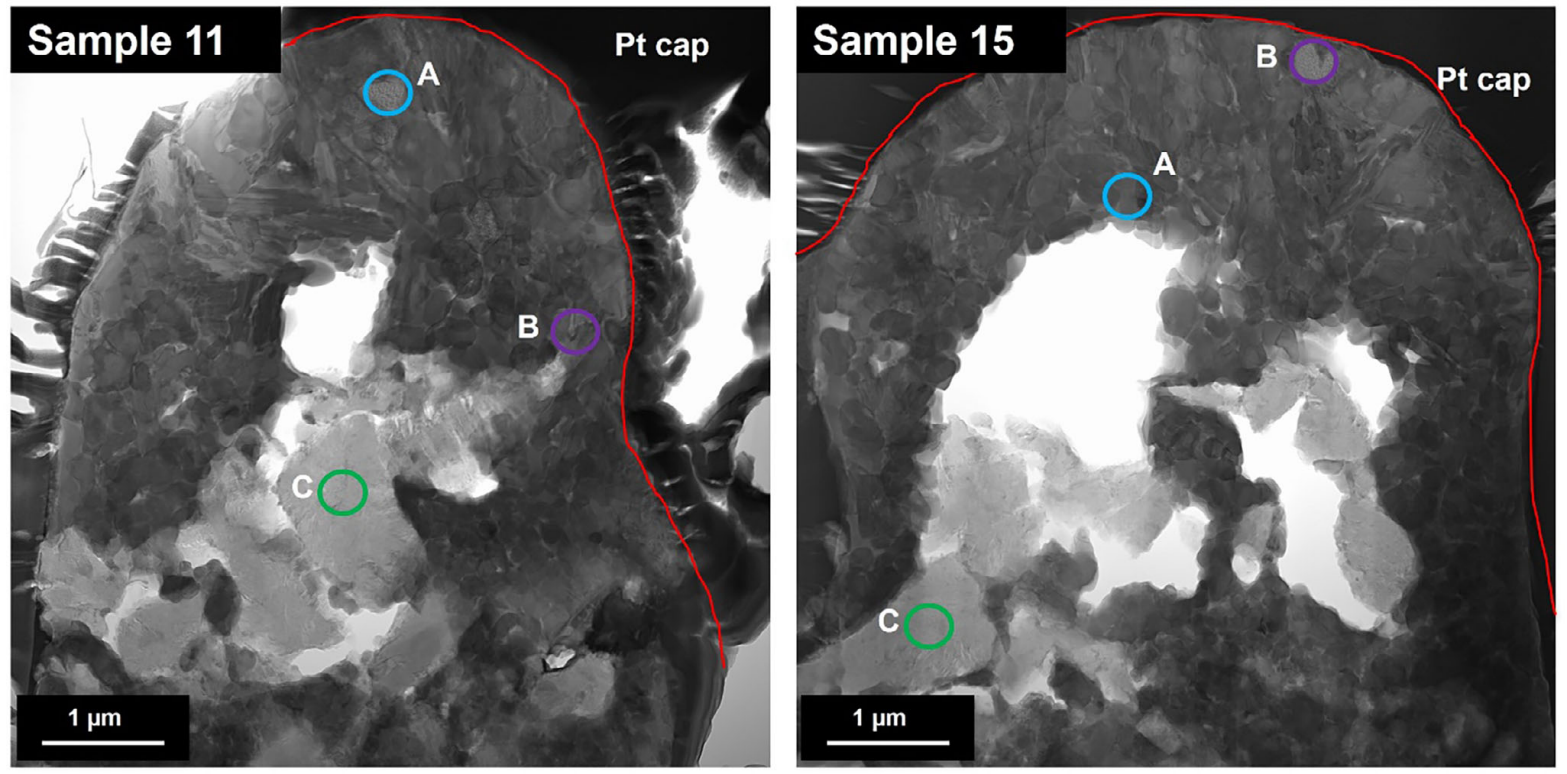
Atomic % STEM EDX Maps for Sample 11 a) and for Sample 15 b).

The STEM images show that the nodules consist of a high aspect ratio nodule ≈4–5 µm in width with a hollow core and wall ≈1–2 µm in thickness. The centre of the nodule consists of boron nitride with a mixed Ti‐containing outer face. The STEM images show that the morphology of the Ti rich phase is modified. In sample 11, acicular rod‐shaped precipitates are observed, while in sample 15 more round equiaxed grains are observed, these findings are consistent with the FIB analysis and suggest differences in heat input and cooling rates, which affected the microstructure. EDS analysis revealed localized redistribution of titanium, boron, and carbon. Boron and titanium were enriched in micro‐agglomerates (Figure 6a,b,e,f), suggesting the formation of new compounds during rapid solidification in air. This is supported by the presence of an oxide layer around the nodules. STEM EDX point spectra were extracted from the STEM EDS data to confirm the compositions of the phases identified (see **Figures** **12** and **13**, and Table 1).

The EDS elemental maps showed a Ti‐rich matrix with areas of boron segregation, strongly indicating the in situ formation of boride compounds, such as TiB or TiB_2_, consistent with previous findings in laser‐processed Ti–B systems.^[^ ^31^ ^]^ As a result of the presented experiments TiC did not form. This is a very stable, hard, and refractory material which typically forms at elevated temperatures (>1500 °C) in vacuum, inert gas, or reducing atmospheres.^[^ ^33^ ^]^ Therefore, processing in air has not favored the formation of this compound. However, a ternary compound (complex phase) based on a Ti (CN) phase has been produced, as supported by the elemental data in Table 1, which shows the composition of the phases formed, particularly under non‐equilibrium conditions typical of laser melting.

Accurate quantification of the microstructure in this case is challenging as the nodules are comprised of overlapping nano size (≈500 nm) grains within a Ti‐rich matrix. Further quantification of light elements, including C, N, and B, is challenging by EDS due to the low energy of the characteristic X‐ray emitted. The use of a windowless detector mitigated some of these uncertainties however, the discrepancies in precise quantification can be accounted for by these differences and additional scattered X‐rays, which contribute to the background Fe, Co, and Cu signals. Despite these challenges the ratios of atomic percentage are consistent with the phases expected. The presence of Ti–C–N phases was also detected. While their exact stoichiometry could not be conclusively determined due to partial amorphization and nanoscale dimensions, their contribution to the overall mechanical performance cannot be entirely dismissed. However, the influence of Ti–C–N phases on mechanical properties appears to be limited, primarily due to their low volume fraction relative to the dominant TiB_2_ and TiN phases. High‐resolution TEM and SAED analyses indicate that these Ti–C–N phases are either nanocrystalline or partially amorphous and are distributed non‐uniformly at the grain boundaries or within the matrix. As such, their direct impact on bulk mechanical properties is difficult to quantify using conventional characterization methods. Moreover, the strengthening mechanisms associated with Ti–C–N, such as dispersion hardening and grain boundary pinning, likely overlap with those already contributed by TiB_2_ and TiN. This overlap further complicates the isolation of their individual effects. Future investigations aimed at detailed quantitative phase analysis (e.g., Rietveld refinement), spatially resolved mechanical testing, and high‐resolution modelling will be valuable for assessing the specific role of Ti–C–N phases in enhancing hardness, strength, or fracture resistance. Nonetheless, based on the current data, it is reasonable to conclude that their contribution is secondary to that of the more abundant and crystalline TiB_2_ and TiN phases.

The atomic percentage data obtained from EDS point analysis (Table 1) should be interpreted with caution, particularly in the case of light elements such as boron and carbon. While several points identified as “TiB” phases show B:Ti atomic ratios deviating from the ideal stoichiometric values for TiB (1:1) or TiB_2_ (2:1), for instance, B:Ti ≈ 45:48 in Sample 11 (Point 2) and ≈ 52:41 in Sample 15 (Point 5), these variations are not unexpected. Quantification of boron by EDS is inherently challenging due to its low atomic number and proximity to the detector's lower energy limit, which results in higher measurement uncertainty. In the present setup, deviations of ±10% are typical for boron‐containing phases. Within this margin of error, the observed compositions remain consistent with TiB‐type or TiB_2_‐type borides, although slight B‐rich or Ti‐rich regions and multiphase mixtures are also possible. This interpretation is further supported by XRD results, which confirm the coexistence of multiple titanium‐based borides and nitrides rather than a single stoichiometric phase.

The relatively high carbon content measured by EDS (≈4–8 at.%) also warrants consideration. Carbon quantification via EDS is subject to substantial error, especially in boron‐rich systems where peak overlaps can occur. The detected carbon may originate from multiple sources, including intrinsic carbon from the TiC binder, residual contamination from sample preparation, or adventitious carbon deposition induced by the electron beam. Nonetheless, the possible role of carbon in the observed phase evolution cannot be entirely excluded. Carbon could contribute to localized compositional fluctuations within the melt pool, influence wetting behavior, or participate transiently in Ti–C–B–N interactions before solidification. Future work should therefore aim to elucidate the influence of carbon more systematically through complementary surface analysis techniques (e.g., XPS or TEM–EELS) and controlled atmosphere experiments to isolate its effects on TiB_2_ and TiN formation.

**Figure 14:**
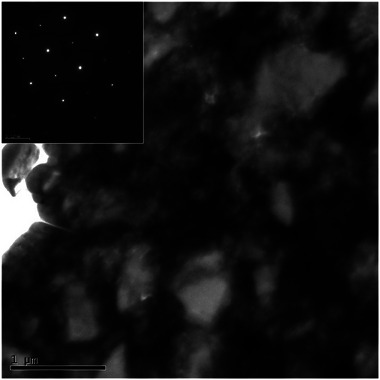
Conventional TEM bright field image of the as‐received microstructure and insert selective area diffraction of the PcBN phase.

HRTEM analysis provided direct crystallographic evidence of nanoscale precipitates formed in the melt pool and interfacial regions. Selective area diffraction from the as‐received sample shows the crystalline phase of cubic Boron Nitride phase (**Figure** **14**).

**Figure 15:**
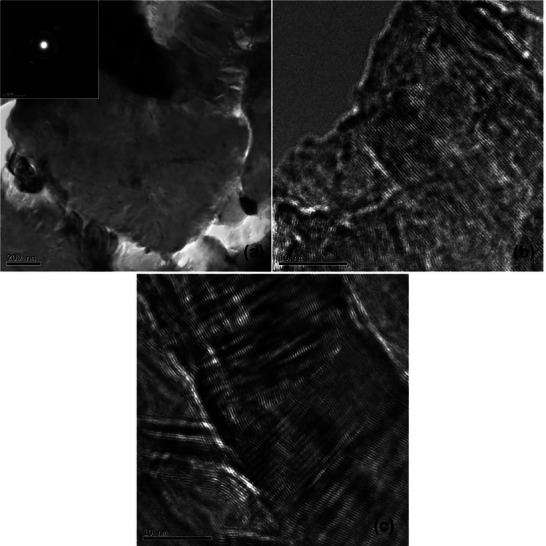
Sample 11 inner surface: ring pattern can be observed in the diffraction pattern a). The SAD aperture used is ≈200 nm diameter, the nano crystalline diffraction pattern b) suggests that the phase formed has many fine crystalline domains less than 200 nm c).

**Figure 16:**
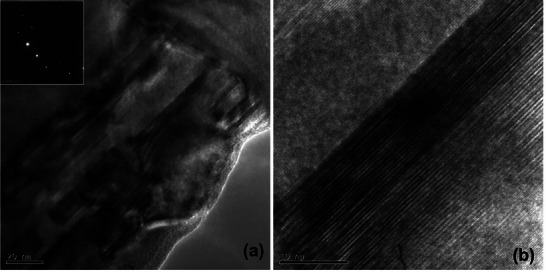
Sample 11 Upper: This image shows the microstructure of the sample corresponding to the upper region of ablation.

**Figure** **15** shows the HRTEM and selective area diffraction (SAD) analysis of the TiB_2_ phase on the inner face of sample 11. The ring patterns with segments of high intensity suggest a nanocrystalline grain structure.^[^ ^34^ ^]^ Lattice‐resolved imaging and selected area electron diffraction (SAED) patterns identified nanocrystalline phases (Figure 15b) with lattice spacings corresponding to TiB_2_, supporting the hypothesis of in situ reaction between Ti and cBN under laser‐induced thermal fields. HRTEM micrographs (Figure 15b,c) show the nanostructure of the formed material with domains on the 10–50 nm scale. **Figure** **16** shows HRTEM images of the microstructure of the sample corresponding to the upper region of ablation. Highly dense twins or stacking faults can be observed in Figure 16a,b. The Selected Area Diffraction (SAD) and HRTEM images reveal that the top surface of the nodules exhibits a noticeably larger grain size compared to the finer crystallites observed at the previously examined internal region, as shown in Figure 15. This grain size disparity suggests that the external surface experienced a different thermal history than the interior during laser processing, likely due to variations in heat dissipation and localized temperature gradients across the nodule structure. These observations indicate that localized laser heating has significantly altered the microstructure of the material's surface. The thermal energy input appears to have promoted both solid‐state and liquid‐phase reactions at the interface between the TiC binder and the cBN hard phase. As a result, new phases have formed, characterized by the development of reinforcing nanostructures. These microstructural changes point to enhanced bonding and potential improvements in mechanical properties, underscoring the critical role of thermal effects and laser parameters in tailoring the surface architecture of such composite materials. These results collectively demonstrate that localized laser heating not only enhances densification but also drives solid‐state and liquid‐phase reactions between the binder TiC and the hard phase cBN, leading to the formation of reinforcing nanostructures.

**Figure 17:**
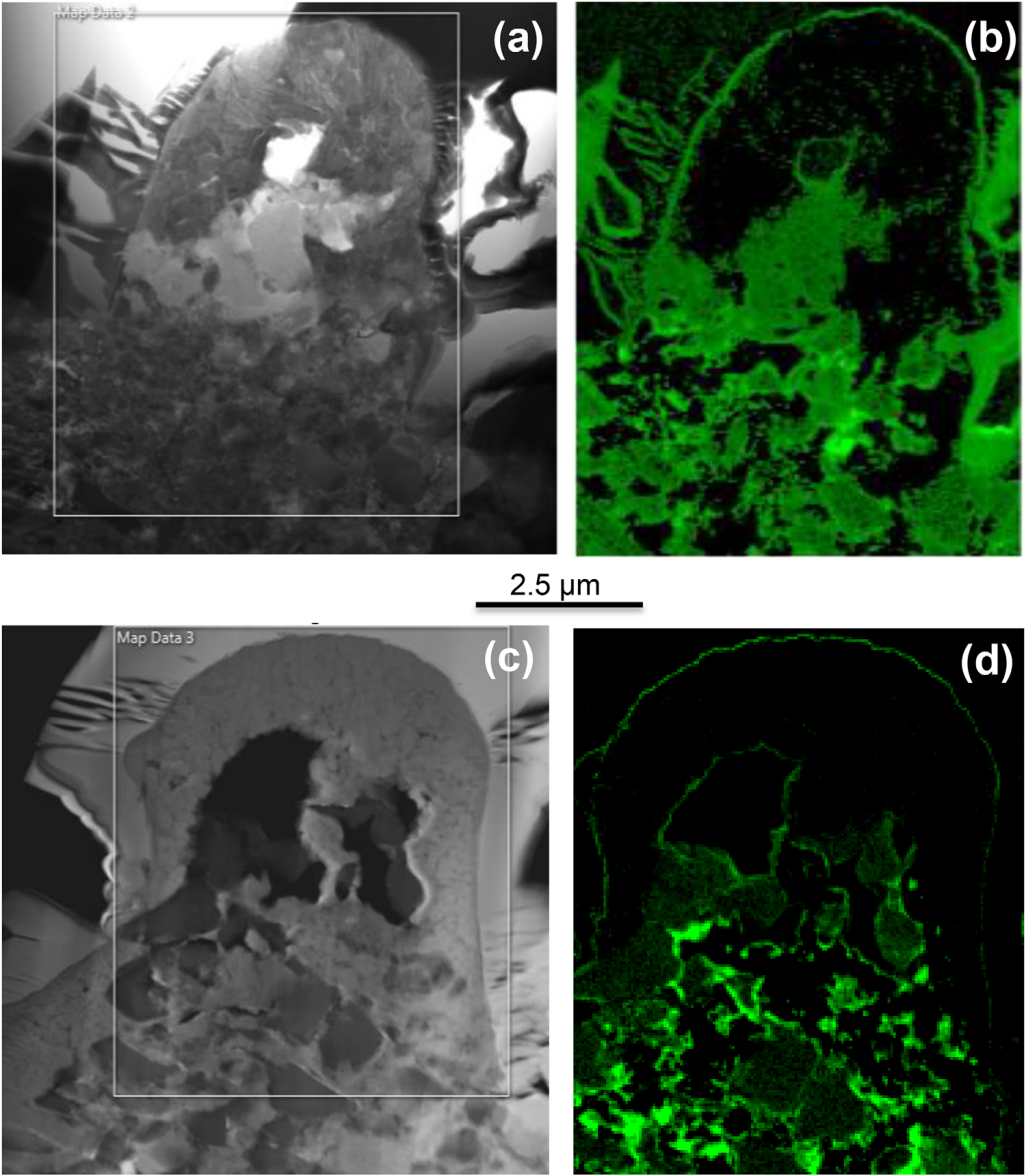
Focused Ion Beam cross sections and EDS mapping of the Oxygen levels in Sample 11 a) and b) and Sample 15 c) and d).

One of the most unexpected and significant findings of this study is the minimal formation of titanium oxides, such as TiO_2_, despite laser processing being performed in ambient air, a condition under which titanium typically oxidises rapidly. This result deviates from the well‐established behavior of laser‐treated titanium systems and suggests that alternative reaction pathways are favored under the specific conditions used here. To better understand this phenomenon, high‐resolution EDS elemental mapping and line scans were performed on FIB‐prepared cross‐sections of the treated regions. These analyses, presented in **Figure** **17**, reveal notable differences in oxygen distribution between Sample 11 and Sample 15. Sample 11 shows more residual oxygen, particularly around laser‐induced bubble features, while Sample 15 exhibits significantly reduced oxygen content in the modified zone. These differences correlate with their respective laser parameters: Sample 11 was treated using short, high‐energy pulses (10 ns, 0.83 GW cm^−^^2^), whereas Sample 15 experienced longer, lower‐energy pulses (130 ns, 0.06 GW cm^−^^2^). The reduced oxidation in Sample 15 may result from several contributing factors. First, the decomposition of cBN under laser irradiation produces reactive boron and nitrogen species that can rapidly react with titanium to form TiB_2_ and TiN, both of which have highly negative Gibbs free energies of formation (−322 and −338 kJ mol^−1^, respectively).^[^ ^35^, ^36^ ^]^ In the local environment created by cBN breakdown, the activities of B and N may exceed that of oxygen, driving the system toward boride and nitride formation rather than oxidation. Second, even if a thin TiO_2_ layer forms initially, it may be removed by subsequent laser pulses through thermal ablation, spallation, or material reflow, thereby continuously exposing fresh titanium for further reaction with B and N. Third, the rapid formation of dense TiB_2_ and TiN layers may act as passivating barriers that suppress further oxygen ingress. Collectively, these mechanisms point to a kinetically driven, self‐organizing process that favors Ti–B–N phase formation over oxidation. This counterintuitive behavior highlights the complex dynamics of laser‐induced reactions in multiphase systems and underscores the potential of cBN as a reactive medium that alters the oxidation kinetics of titanium in air.

#### 2.2.2 Characterization via XRD

**Figure 18:**
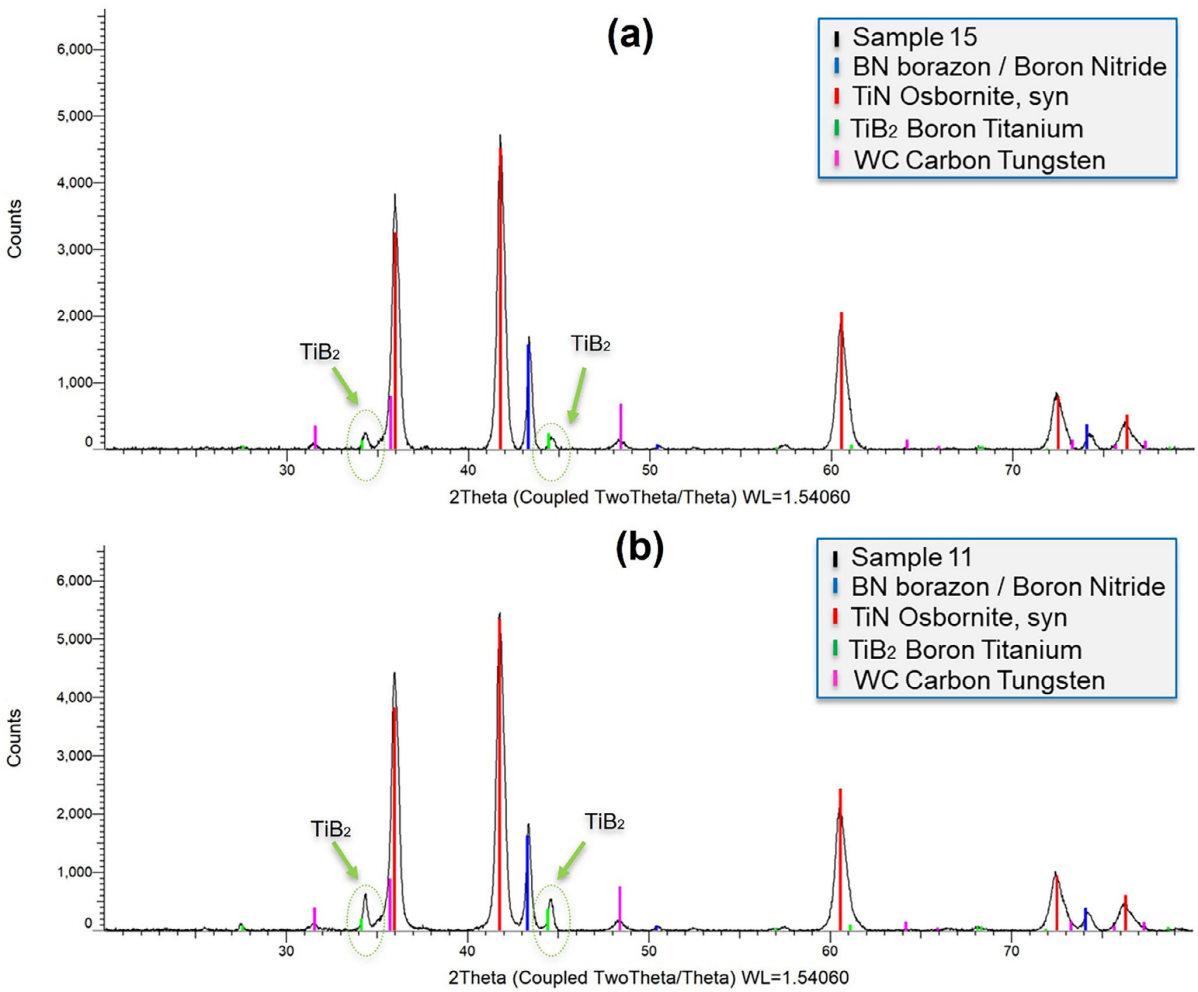
XRD patterns of the specimens under laser surface processing condition a) in Sample 15 and b) in Sample 11.

Phase identification of the samples was conducted using a Bruker D2 X‐ray Diffractometer. Each sample was scanned over a 2θ range of 20° to 80°, with a step size of 0.2° and a step time of 1.0 second per increment, resulting in a total scan duration of ≈3240 s. The XRD patterns (**Figure** **18**) revealed the presence of at least four distinct crystalline phases in all samples: boron nitride (BN), titanium nitride (TiN), titanium diboride (TiB_2_), and tungsten carbide (WC). A comparison of the diffraction data shows that Sample 11 consistently exhibits slightly higher peak intensities than Sample 15 for the same phases. This increase in intensity may indicate a higher degree of crystallinity or a greater relative abundance of these phases in Sample 11. In X‐ray Diffraction (XRD), sample 11 showed higher peak intensities compared to sample 15 generally indicating a greater number of aligned atoms leading to constructive interference of the diffracted X‐rays.^[^ ^37^, ^38^ ^]^ This can be due to several factors, including increased crystallinity, larger crystal size, or a higher concentration of a specific phase within the material. In our case diffraction pattern shown in Figure 15 shows a greater crystallinity for sample 11, both for TiB_2_ and TiN, which corroborates the increased peak intensity shown in the XRD results. Several prior investigations have explored the fabrication of Ti–cBN composite structures via selective laser melting (SLM). For instance, Mynasian et al.^[^ ^26^ ^]^ reported that the addition of Ti enhances laser energy absorption and promotes densification in the presence of cubic boron nitride (cBN). They suggested that localized melting might induce chemical reactions between Ti and cBN, potentially leading to the in situ formation of titanium diboride (TiB_2_). However, their study did not explicitly confirm the formation of TiB_2_ through phase analysis, such as X‐ray diffraction (XRD), nor did it include detailed microstructural characterization. In contrast, other studies, such as those by Hussain et al.^[^ ^39^ ^]^ and Bathala et al.^[^ ^40^ ^]^ have directly observed the in situ precipitation of TiB_2_ during laser‐based processing techniques, including laser powder bed fusion (L‐PBF) and laser cladding. In Ti–32Nb–7Zr–5Ta–TiB_2_ alloy systems, laser processing facilitated the formation of TiB or TiB_2_ nanoparticles with whisker or needle‐like morphologies, particularly in interdendritic regions. Similarly, Promakhov et al.^[^ ^43^ ^]^ reported that laser cladding of Ni‐based alloys could promote the formation of TiB_2_ inclusions, with their presence and morphology strongly influenced by laser parameters and the composition of the feedstock powders. Collectively, these findings confirm that under rapid solidification conditions typical of laser processing, titanium and boron can readily react to form nanoscale TiB_2_. While previous laser sintering efforts involving pre‐packed layers of cBN and metal binder powders (e.g., bronze or Ni) primarily aimed to improve bonding and densification, they did not provide direct evidence of TiB_2_ formation. In contrast, our present study demonstrates for the first time that localized high thermal gradients during laser sintering can trigger in situ reactions when titanium‐based precursors are incorporated into the binder system. The peaks identified in the XRD analysis for TiB_2_ match the characteristic X‐ray diffraction (XRD) pattern of titanium diboride (TiB_2_) reported in previous studies. The most prominent diffraction peaks typically appear at approximately 2θ ≈ 27.7°, 34.3°, 41.8–44.6°, and 61.3°, corresponding to the (001), (100), (101), and (110) planes, respectively. These reflections are indicative of the highly crystalline nature of TiB_2_.^[^ ^42^ ^]^ In a study by Mollica et al. (1999), TiB_2_ peaks were observed at 2θ = 44.5° and 61.5°, aligning closely with standard reference data.^[^ ^43^ ^]^ These slight shifts in peak positions can be attributed to lattice strain, particle size effects, and instrumental factors, but the overall agreement across different sources confirms the reliability of these reflections as fingerprints of hexagonal TiB_2_. In our case, how it can be seen in Figure 18, the green peaks corresponding to the TiB_2_ are evident for 2θ = 34.5° and 2θ = 44.5°. The first one (34.5°) is well within the range of a TiB_2_ in the (100) plane, which, from existing data, is 34.3°–34.6°.^[^ ^42^, ^43^ ^]^ The second one is well within the range of a TiB_2_ in the (101) plane, which from existing data, is 41.8°–44.6°,^[^ ^42^, ^43^ ^]^ corroborating the synthesis of TiB_2_ in our study.

### 2.3 Formation Mechanism of TiB_2_ and TiN Nanoclusters

Under nanosecond laser processing via pulsed fiber lasers, TiB_2_ is formed in situ when titanium is exposed to a boron source (such as elemental boron or boron‐containing compounds like BN, B_4_C, or amorphous B). The Ti–B reaction is exothermic and, once initiated at high temperatures (typically above 1200 °C), can rapidly proceed.^[^ ^45^ ^]^ Laser irradiation easily exceeds this threshold, especially under ns pulses, which can locally raise temperatures to 2000–3000 °C or more.^[^ ^46^, ^47^ ^]^ Previous research^[^ ^47^ ^]^ demonstrated formation of TiB_2_ in laser grinding of PcBN, however in this study we were able to achieve TiB_2_ formation for the first time through nanosecond laser processing. If Ti‐coated PcBN (Ti + cBN) materials are exposed to laser melting, this generates the following reactions: i) Ti dissolves and interacts with boron from cBN; ii) TiB_2_ precipitates during rapid cooling of the melt, forming embedded reinforcing phases. In our experiments, TiB_2_ formation is demonstrated by XRD analysis and FIB/EDS analysis (presented in Figure 18 and Table 1, respectively). TiB_2_ is a hard, thermally stable compound with excellent wear resistance. TiB_2_ typically forms through solid‐state diffusion at temperatures above ≈1400 °C; however, during laser processing of PcBN, it is expected that this might form at lower temperatures due to liquid or interfacial reactions.^[^ ^41^ ^]^ Titanium reacts with boron as shown in Equation (1):

(1) $$Ti+2B\to TiB_{2}Ti+2B\to TiB_{2}$$

Due to cBN being present in the material at 50 wt%, heating the material leads to decomposition or chemical exchange reactions, particularly involving titanium. When subjected to high temperatures, typically between 1200 and 1600 °C,^[^ ^44^, ^48^ ^]^ as encountered during sintering or laser processing, titanium reacts with BN to form titanium diboride (TiB_2_) and titanium nitride (TiN).^[^ ^50^ ^]^ This reaction can be represented as in Equation (2):

(2) $$2Ti+BN\to TiB_{2}+TiN_{2}Ti+BN\to TiB_{2}+TiN$$

**Figure 19:**
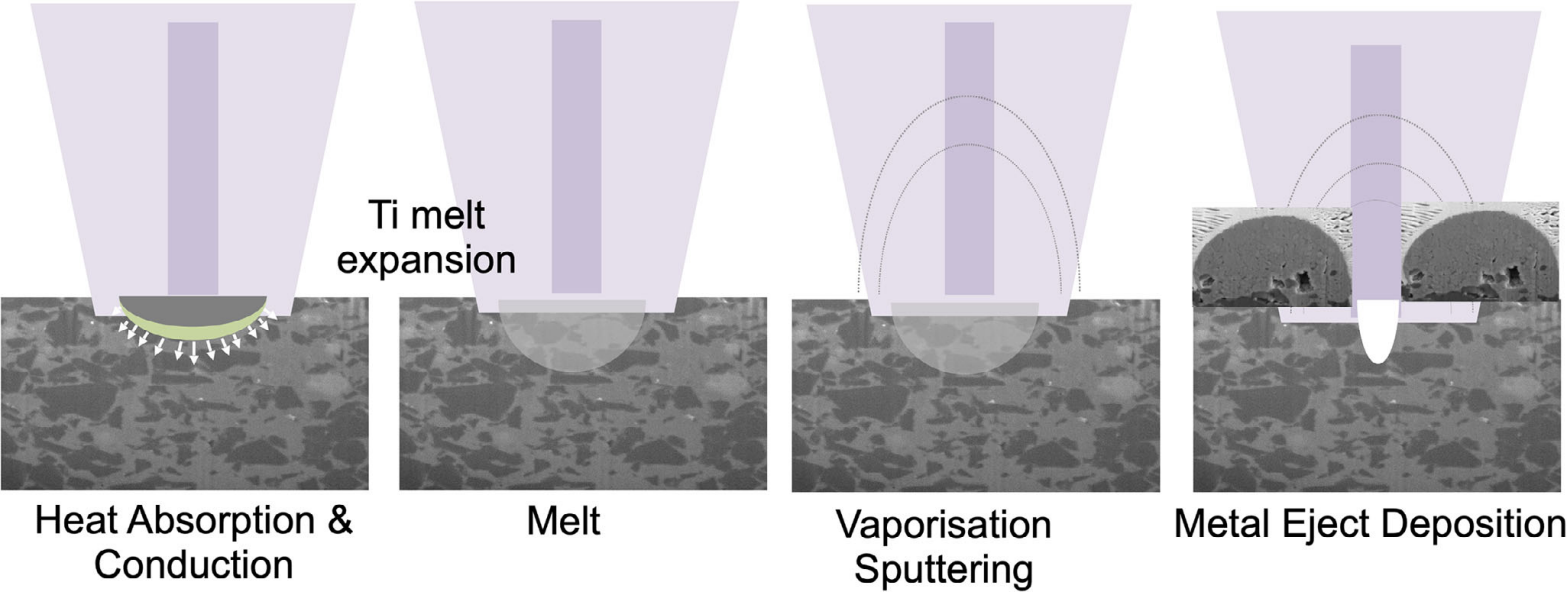
Schematic diagram of spherical micro‐agglomerates formation.

This in situ transformation occurs as titanium extracts boron and nitrogen directly from BN, which serves as a combined source of both elements. The result is the simultaneous formation of two thermodynamically stable ceramic phases, TiB_2_ and TiN, within the evolving microstructure. Since cubic boron nitride is present in 50 wt%, this concentration is significantly higher than the titanium and carbon binder alone, thus favoring TiB_2_ and TiN formation. From a thermodynamic standpoint, the formation of both TiB_2_ and TiN is strongly driven by negative Gibbs free energy (Δ*G*) values across the processing temperature range. According to the Gibbs free‐energy criterion, a thermodynamic driving force exists when Δ*G* < 0 as defined in Equation (3):

(3) $$\Delta G\left(T\right)=\Delta H-T\Delta S$$

where Δ*H* and Δ*S* are the enthalpy and entropy changes. The reaction Ti+2B→TiB_2_ exhibits a highly negative ΔG° (approximately –320 to −360 kJ mol^−1^ between 1000 and 1600 °C), confirming its spontaneous nature even under limited diffusion.^[^ ^35^, ^36^ ^]^ Similarly, the formation of TiN via Ti+1/2 N_2_ →TiN or from BN sources maintains a Δ*G*° of roughly −290 kJ mol^−1^ at 1500 °C, which further decreases with temperature. The thermodynamic favorability of both compounds increases at elevated temperatures since their formation enthalpies (ΔH°) are large and negative, while the entropy change (ΔS°) remains small or slightly negative, ensuring that the –TΔS term does not overcome the exothermic enthalpy component. Consequently, even in the transient thermal environment of nanosecond laser irradiation, characterized by extreme temperature spikes and rapid quenching, both reactions proceed spontaneously once initiated. It is also noteworthy that TiB_2_ formation generally precedes TiN formation under similar conditions, owing to its more negative Gibbs free energy and stronger Ti─B bond energy compared to Ti–N. However, in the Ti–BN system, nitrogen becomes liberated concurrently as boron is extracted from BN, leading to nearly simultaneous precipitation of both TiB_2_ and TiN during cooling.^[^ ^45^, ^46^ ^]^ Under rapid solidification, TiB_2_ tends to nucleate heterogeneously at the Ti–B–N melt interfaces, while TiN often forms as finer dispersed particles due to its slightly lower nucleation energy barrier under nitrogen supersaturation. Previous work^[^ ^48^ ^]^ explained the in situ formation mechanism of titanium carbide (TiC) during selective laser melting (SLM) of a Ti–Ni–C powder mixture. While formation of TiC occurs through a dissolution–precipitation process that takes place within the localized molten pool generated by the laser, these findings corroborate our hypothesis that the intense laser energy melts the TiC binder as well as the boron nitride, creating a molten matrix in which carbon becomes readily dissolved, resulting in a Ti–B–N liquid solution. As the melt begins to cool and solidify, the solubility of carbon in the titanium‐rich matrix decreases.^[^ ^49^ ^]^ When the carbon becomes supersaturated in the melt, it precipitates out in the form of stoichiometric or near‐stoichiometric TiN and TiB_2_. The solidification of TiN and TiB_2_ follows one primary route: in areas where partially melted TiC particles are present, the precipitation occurs through epitaxial growth along the particle boundaries. The thermodynamic and kinetic conditions within the molten pool play a crucial role in governing the size, distribution, and morphology of the resulting phases. The formation of regrown regions consisting of titanium‐based compounds (TiB_2_ and TiN) can be attributed to thermally induced phenomena occurring during laser processing. Formation of TiB_2_ in PcBN composites post laser processing was previously demonstrated during laser grinding.^[^ ^46^ ^]^ The mismatch in thermal expansion coefficients between the binder phase (TiC) and the hard ceramic grains (cBN) leads to localized temperature gradients and, consequently, variations in surface tension across the molten regions.^[^ ^49^, ^50^ ^]^ When the scan rate is too high or the energy input too low, the melt exhibits higher viscosity and surface tension. This results in poor melt flow and incomplete filling of gaps between particles, leading to larger pores and the formation of spherical metallic agglomerates, or "balls." While processing, the system exhibits the coexistence of two or more liquid phases, which generate spatial gradients in surface tension. These gradients induce fluid motion driven by the Marangoni effect, where molten material flows from regions of lower to higher surface tension. This thermocapillary convection results in the characteristic cellular flow patterns (Bénard–Marangoni convection)^[^ ^51^, ^52^, ^53^ ^]^ observed in the regrown microstructures (Figure 1c). In the subsequent cooling stage, which is a very rapid air quenching, the induced micro/nano‐structural features are preserved, as there is no reversion to the previous solid state. As the molten alloy cools, nucleation of TiN and TiB_2_ begins, with tiny carbide crystals starting to form and grow throughout the liquid metal. This is corroborated by XRD analysis (Figure 18), which clearly shows the peaks of these two compounds. What makes this process particularly interesting is the shape that the TiB_2_ and TiN particles assume. Rather than forming faceted or dendritic structures, as previously demonstrated in relevant research,^[^ ^45^, ^51^ ^]^ the particles develop into smooth, spherical shapes. At higher fluence levels, the ablation threshold is exceeded, and smooth vaporization transitions into an explosive boiling regime (**Figure** **19**). This phase change is accompanied by the ejection of microparticles ranging in size from 0.5 to 10 µm. The formation mechanism of the spheres can be explained in four main stages: i) particles absorb laser energy through bulk‐ and powder‐coupling mechanisms;^[^ ^27^ ^]^ ii) surface melting occurs first, followed by heat conduction inward; iii) liquid formation and flow are governed by laser power and scan speed; iv) insufficient liquid from low power/high speed raises viscosity, impairs rheology, and promotes ball formation. This agrees with previous work^[^ ^54^ ^]^ where balling as a defect appearing in direct metal laser sintering was investigated, and it was demonstrated that high scan speed produces numerous small (≈10 µm) spheres from melt splashes, caused by capillary instability in the molten pool. When PcBN composites undergo a thermal process such as laser ablation, a variety of solid‐state and gas–solid reactions occur, which depend on the specific processing conditions such as pressure, phase composition, and temperature. This results in a series of chemical reactions driven by thermal gradients, which aid formation of various stable ceramic phases. Due to the presence of cBN hard grains, titanium reacts with both elements (boron and nitrogen) to form TiB_2_ and TiN through in situ reactions. Carbon does not directly react with BN under typical conditions, but the presence of titanium enables cross‐element reactions. The atmosphere plays a crucial role in determining the reaction pathway. Since the process is in air, one would expect titanium to oxidise to TiO_2_, suppressing nitride formation. However, in our experiments, we demonstrated the formation of TiB_2_ and TiN in air for the first time, despite these compounds typically forming in vacuum or inert atmospheres.^[^ ^33^ ^]^ This observation further reinforces the strong thermodynamic stability (Δ*G*° ≪ 0) of TiN and TiB_2_, which can outcompete oxidation under short laser exposure times where oxygen diffusion is limited. Overall, the thermal behavior of this system is governed by the strong affinity of titanium for carbon, boron, and nitrogen, leading to the development of hard, refractory compounds with tuneable microstructures. The negative Gibbs free energies of formation for TiN and TiB_2_ across a wide temperature range confirm that their synthesis is thermodynamically spontaneous, while the high localized energy and rapid quenching provided by nanosecond laser pulses kinetically stabilize their fine, spherical morphologies observed experimentally.

## 3 Conclusion

This study presents a novel laser‐based manufacturing method for the in situ formation of spherical and woven‐like TiN and TiB_2_ micro and nanostructures directly from a solid PcBN substrate. Unlike traditional approaches requiring external feedstock, this technique leverages controlled laser ablation to drive localized chemical reactions and surface transformations, enabling a new pathway for microstructural engineering in superhard materials. Under the selected nanosecond pulsed fiber laser conditions, the interaction between the laser beam and the Ti‐coated PcBN surface occurs within a high‐fluence, short‐pulse regime. At these pulse durations (45–220 ns), the absorbed laser energy produces extremely high local heating rates and steep thermal gradients, resulting in partial surface melting, localized vaporization, and re‐deposition of ejected material. Complete melt pool development, as typically observed in Laser Powder Bed Fusion (LPBF) or continuous‐wave laser processing, does not occur under these transient conditions. Instead, the process is governed by rapid cyclic heating and cooling, where surface atoms are momentarily excited to near‐boiling temperatures before resolidifying within microseconds. This leads to the formation of fine‐scale microstructural features, spherical re‐solidified particles, and re‐deposited layers consistent with vapor condensation phenomena. The localized melt regions experience strong Marangoni‐driven flow and capillary instabilities, influencing the observed morphology and phase distribution. The thermal response and phase transformations are thus dominated by the non‐equilibrium nature of nanosecond laser irradiation, where temperature, diffusion, and cooling rates are all significantly higher than in conventional thermal treatments. These extreme conditions promote in situ reactions between titanium, boron, and nitrogen species, leading to the spontaneous formation of TiB_2_ and TiN within the near‐surface region. The in situ formation of TiB_2_ and TiN during nanosecond laser processing of Ti‐coated PcBN is both thermodynamically and kinetically favored. The strongly negative Gibbs free energies of formation for these compounds ensure spontaneous synthesis once high local temperatures are achieved, while the rapid heating–cooling cycles of laser irradiation promote fine, uniformly distributed phases. These conditions not only stabilize TiB_2_ and TiN against oxidation even in ambient atmosphere but also lead to the development of dense, spherical morphologies that may enhance the hardness and wear resistance of the composite. Overall, the combined thermodynamic stability and kinetic control provided by laser processing enable the tailored formation of refractory titanium‐based ceramics within the PcBN matrix.

For the first time, laser ablation is demonstrated as a dual‐function tool capable of simultaneously modifying both the chemical composition and geometric features of cutting tools. This challenges the conventional separation between material/coating design and tool geometry optimization, particularly in applications such as chip breaker formation, opening possibilities for multifunctional tool surfaces engineered at micro and nano‐scales. The direct formation of spherical TiN and TiB_2_ nanoparticles under laser‐induced combustion synthesis conditions is attributed to a combination of in situ chemical reactions, rapid cooling, and surface tension regulation. Reverse thermal hysteresis, driven by differences in thermal conductivity among TiC, BN, and the surrounding melt, plays a key role in local temperature distribution and nucleation behavior. Additionally, carbon diffusion kinetics and concentration gradients critically influence the reaction rates and resulting microstructural features. Overall, these findings confirm that under the rapid solidification and high thermal gradient conditions typical of laser processing, titanium and boron can readily react to form nanoscale TiB_2_. This contrasts with prior laser sintering efforts using pre‐packed powder layers, which did not provide direct evidence of such phase formation. The creation of the synthesized structures emerges from a complex interplay of thermal dynamics and partial melting, underlining the unique capabilities of laser surface engineering in tailoring both material chemistry and functional geometry in a single processing step.

This study demonstrates, for the first time, the in situ formation of TiB_2_ and TiN during nanosecond laser processing of Ti‐coated PcBN, driven by strongly negative Gibbs free energies and facilitated by localized high‐temperature reactions within the laser‐induced melt pool. The experimental evidence confirms that both phases can form spontaneously under transient thermal conditions, even in ambient air. However, while the thermodynamic analysis supports the feasibility of these transformations, the precise kinetics and sequence of TiB_2_ and TiN nucleation remain uncertain due to the extreme temporal and spatial gradients inherent to laser–matter interactions. To conclusively identify the dominant formation pathways, whether through solid–liquid interfacial reactions, vapor‐phase condensation, or dissolution, precipitation mechanisms, future work should incorporate in situ time‐resolved diagnostics (e.g., high‐speed spectroscopy or synchrotron‐based X‐ray diffraction) alongside advanced thermal‐fluid modelling of the laser melt pool. In addition, future studies should include systematic validation of the resulting coatings’ mechanical performance, particularly hardness, adhesion, and wear resistance, to correlate the observed microstructural evolution with functional properties. Such combined experimental and modelling efforts will be essential for explaining the dynamic phase evolution and optimizing processing parameters for controlled microstructural development in Ti–B–N systems.

## 4 Experimental Section

### Materials

A commercial grade of PCBN was used for the research, a low cBN content PCBN DCC500 (CBN, average grain size 1.5 µm; TiC binder ≈50 vol. %) directly synthesized on a Tungsten Carbide backing (Element Six Ltd). The average grain size of this composite is 1.5 µm and it is an ideal material for cold work tool steels, valve seat alloys and high strength cast irons.^[^ ^55^ ^]^ Some of the thermal properties of the main binder compositions, TiC for the DCC500 are shown in **Table** **2**.

**Table 2:** Properties of the main binder compound for DCC500.^[^ ^56^, ^57^ ^]^

Properties	Titanium carbide (TiC)
Density [kgm^−3^]	4930
Enthalpy [kJ mol^−1^]	–
Melting Temperature [K]	3430
Vaporisation Temperature [K]	5090
Thermal Conductivity [W m^−1^ K^−1^]	30.93 (at 300 K) 5.64 (at 1273 K)
Specific Heat at Constant Pressure [J kg^−1^ K^−1^]	750 (at 573 K) 820 (at 873 K) 878 (at 1273 K)
Thermal Expansion Coefficient [K^−1^]	9.2 to 9.8 × 10^−6^

### Laser Surface Processing

The laser processing was carried out using a 70 W Innolas MMS single mode SPI fibre laser with a wavelength of 1060 nm. The computer numerically controlled (CNC) laser machine, operating in pulse mode, delivered pulses through direct modulation of the seed laser, allowing for programmed waveforms. These waveforms were the outcome of optimizing peak power at a specific pulse repetition rate. The pattern movement for the laser was controlled by a series of mirrors inside the galvanometer head. The change in beam incident angle is negligible (less than 0.2°). Laser parameters and ranges for the experiments are reported in Table 1. Laser energy density, also known as irradiance, was calculated using Equation (4):

(4) $$PL_{i}=\frac{4P_{m}}{\pi \tau d_{i}^{2}f}\;\left[W\;\;cm^{-2}\right]\;\;i=1,2\text{…}5$$

where *d_i_* is the beam spot diameter [cm], *f* is the laser pulse frequency [Hz], *P_m_* is the average laser power [W], *τ* is the laser pulse duration (µs). To enable calculation of *PL_i_* the beam spot diameter, *d_i_*, was measured based on a method developed in previous work.^[^ ^58^ ^]^ To obtain *d_i_* an investigation into spot diameter at different focal heights was carried out. The beam diameter at the focal points was measured to be ≈29 µm.

A fit screening design was first implemented to identify the most influential process parameters affecting surface topography and microstructure. Five parameters were investigated (**Table** **3**) laser intensity (30–80%, 5 levels), repetition frequency (35–170 kHz, 4 levels), feed speed (400–1000 mm s^−1^, 7 levels), pulse duration (10–200 ns, 4 levels), power density (0.04–1.71 GW cm^−2^, 8 levels). The power density was also reported; however, this is a variable dependent on intensity. This factorial design resulted in 17 experimental conditions, which were each repeated three times to ensure statistical robustness. A second experimental phase was then conducted to gain a deeper understanding of the thermo‐mechanical phenomena occurring at various feed speeds, pulse durations, and frequencies, focusing on their influence on surface roughness and morphological formation. In this stage, a Taguchi array was adopted, varying pulse duration, feed speed, and frequency across 3, 4, and 6 levels (**Table** **4**). Each experimental condition in this set was also repeated three times for reproducibility.

**Table 3:** Laser processing parameter range.

Process parameters	Range experiments 1–17
Intensity I [%]	30‐50‐60‐70‐80
Repetition frequency f [kHz]	35‐48‐105‐170
Feed speed FS [mm s^−1^]	400‐500‐600‐700‐800‐900‐1000
Pulse duration τ [ns]	10‐30‐130‐200
Power density [GW cm^−2^]	0.0412‐0.0544‐0.0634‐0.0821‐0.4171‐0.5720‐0.8244‐1.7161

**Table 4:** Laser parameter range used in the various experiments.

Experiment number		Frequency [kHz]	Feed speed [mm s^−1^]	Pulse duration [ns]	Energy Density [GW cm^−2^]	Focal distance [mm]
10	A1	170	400	200	0.0412	61.5
	A2	170	500	200	0.0412	61.5
7	A	170	400	130	0.0544	62.5
	B	170	400	130	0.0634	62.5
	C	170	400	130	0.0725	62.5
8	A	170	400	200	0.0353	61.5
	B	170	400	200	0.0412	61.5
	C	170	400	200	0.0471	61.5
11	A1	35	400	10	1.7161	62.5
	A2	35	400	30	0.5720	62.5
	A3	48	400	30	0.4171	62.5
	B1	48	1000	30	0.4171	62.5
	B2	105	400	130	0.0821	62.5
	C1	170	400	10	0.8244	62.5
	C2	170	400	10	0.8244	62.5
	C3	170	1000	10	0.8244	62.5
12	A1	35	400	10	1.7161	61.5
	A2	35	400	30	0.5720	61.5
	A3	48	400	30	0.4171	61.5
	B1	48	1000	30	0.4171	61.5
	B2	105	400	130	0.0821	61.5
	C1	170	400	10	0.8244	61.5
	C2	170	800	10	0.8244	61.5
	C3	170	1000	10	0.8244	61.5
13	B1	35	600	200	0.2002	61.5
	B2	48	600	200	0.1460	61.5
	B3	100	600	200	0.0701	61.5
	B4	150	600	200	0.0467	61.5
	B5	170	600	200	0.0412	61.5
15	A	170	400	130	0.0544	61.5
	B	170	400	130	0.0634	61.5
	C	170	400	130	0.0725	61.5
16	A1	170	400	200	0.0412	61.5
	A2	170	500	200	0.0412	61.5
	A3	170	600	200	0.0412	61.5
	A4	170	700	200	0.0412	61.5
	A5	170	800	200	0.0412	61.5
	A6	170	900	200	0.0412	61.5
	A7	170	1000	200	0.0412	61.5

### Material Characterization—Metrological Analysis

A 3D white light interferometer (Alicona Infinite Focus) was employed to analyze the surface topography of laser‐processed areas both before and after testing, with a vertical resolution of 110 nm and a lateral resolution of 2.13 µm. In accordance with ISO 13565, Abbott‐Firestone curves were generated for each sample to extract the 2D surface roughness parameter, Ra. Additionally, based on ISO 25178, Abbott–Firestone curves were plotted for the entire surface area, allowing the calculation of the corresponding 3D roughness parameter, Sa.^[^ ^59^ ^]^

### Morphological Characterization and Subsurface Integrity Evaluation

All laser‐processed samples were cleaned in a water‐filled ultrasonic bath for 30 min to remove surface debris. Imaging and chemical composition analysis were performed using a JEOL Zeiss Scanning Electron Microscope (SEM) equipped with energy‐dispersive X‐ray spectroscopy (EDS). SEM images were captured at magnifications of ×7500 and ×20000 for both as‐received and laser‐processed regions to facilitate comparative analysis. Cross‐sectional analysis of the laser‐treated areas was conducted using a Nova 600 NanoLab Ga DualBeam focused ion beam (FIB) system. Before milling, a protective platinum layer (2–10 µm thick) was deposited to prevent subsurface damage. The exposed cross‐sections were subsequently examined via EDS to assess compositional changes. Phase identification of potential oxides was carried out using X‐ray diffraction (XRD) with a D2 Phaser diffractometer (Bruker AXS, Karlsruhe, Germany), employing Cu Kα radiation (*λ* = 1.54054 Å) at 30 kV and 10 mA. Diffraction data were collected over a 2θ range of 20°–100° with a step size of 0.02°. Data analysis was performed using DIFFRAC.EVA 6.1 software (Bruker AXS, Karlsruhe, Germany). Following FIB‐SEM lift‐out samples were analyzed by TEM using a Tecnai F20 FEG‐TEM, equipped with Oxford instruments windowless EDS detector, conventional high‐resolution TEM micrographs were collected with a Gatan CCD detector.

## Conflict of Interest

The authors declare no conflict of interest.

## Data Availability

The data that support the findings of this study are available from the corresponding author upon reasonable request.

## References

[1] J. Shackelford , Introduction to Materials Science for Engineers, 8th ed, Pearson, London, 2015.

[2] M. Pacella , Doctoral Dissertation, University of Nottingham, Nottinghamshire, NG7 2RD 2014, https://eprints.nottingham.ac.uk/27730/1/Pacella%20Manuela%20PhD%20Final%20Thesis.pdf.

[3] T. Burchell , Carbon Materials for Advanced Technologies, Elsevier, Amsterdam, 1999.

[4] K. E. Hazzan , M. Pacella , Manufact. Lett. 2022, 32, 87.

[5] M. Pacella M , P. W. Butler‐Smith , D. A. Axinte , M. W. Fay , J. Mater. Process. Technol. 2014, 214, 1153.

[6] M. Pacella , V. Nekouie , A. Badiee , J. Mater. Process. Technol. 2019, 266, 311.

[7] M. Pacella , A. Badiee , Proc. CIRP 2022, 113, 599.

[8] M. Pacella , D. A. Axinte , P. W. Butler‐Smith , P. Shipway , M. Daine , C. Wort , J. Manuf. Sci. Eng. 2016, 132, 021001.

[9] C. Daniel , S. Ostendorf , S. Hallmann , C. Emmelmann , J. Laser Appl. 2016, 28, 012001.

[10] S. Kalpakjian , S. R. Schmid , K. S. V. Sekar , Manufacturing Engineering and Technology, 2014, 7th ed., Pearson Education, London, UK.

[11] M. G. Warhanek , J. Pfaff , L. Meier , C. Walter , K. Wegener , In Laser‐based Micro‐and Nanoprocessing X, SPIE, Bellingham, Washington, 2016, 328.

[12] A. A. Siddiqu , A. K. Dubey , Opt. Laser Technol. 2020, 134, 106619.

[13] S. Gräf , K. Clemens , F. A. Müller , Materials 2017, 10, 933.28796180

[14] G. Byrne , D. Dornfeld , B. Denkena , CIRP Ann. 2003, 52, 483.

[15] A. Neves , M. H. Nazaré , Properties, Growth and Applications of Diamond, IET, London, 2001.

[16] D. Kalyanasundaram , A. Schmidt , P. Molian , P. Shrotriya , J. Manuf. Sci. Eng. 2014, 136, 041001.

[17] D. Kalyanasundaram , P. Molian , P. Shrotriya , J. Manuf. Process. 2012, 14, 336.

[18] R. Fedorov , F. Lederle , M. Li , V. Olszok , K. Wöbbeking , Chem. Eur. Online Soc. 2021, 86, 1231. 10.1002/cplu.202100118 33960734

[19] W. M. Daoush , T. S. Alkurahiji , A. D. Alshammri , Materials 2021, 14, 7906.34947501

[20] A. Gubernat , P. Klimczyk , P. Pasierb , P. Rutkowski , K. Kornaus , R. Lach , J. Kulczyk‐Malecka , J. Morgiel , J. Eur. Ceram. Soc. 2025, 45, 117393.

[21] S. Zhong , C. Chen , Z. Li , Y. Liu , B. Liu , Y. Wu , J. of Materi Eng and Perform 2020, 29, 3784.

[22] J. Rao , R. Cruz , K. J. Lawson , J. R. Nicholls , Diamond Relat. Mater. 2004, 13, 2221.

[23] M. Das , K. Bhattacharya , S. A. Dittrick , C. Mandal , V. K. Balla , T. S. Sampath Kumar , A. Bandyopadhyay , I. Manna , J. Mech. Behav. Biomed. Mater. 2014, 29, 259. 24121827 10.1016/j.jmbbm.2013.09.006

[24] H. Sahasrabudhe , J. Soderlind , A. Bandyopadhyay , J. Mech. Behav. Biomed. Mater. 2015, 53, 239. 26344856 10.1016/j.jmbbm.2015.08.013

[25] M. Wen , B. Jiang , X. Duan , D. Xiang , Coatings 2025, 15, 815.

[26] T. Minasyan , L. Liu , S. Aydinyan , M. Antonov , I. Hussainova , Key Eng. Mater. 2019, 799, 257.

[27] A. Simchi , H. Asgharzadeh , Mater. Sci. Technol. 2004, 20, 1462.

[28] W. Wang , J. Ning , S. Y. Liang , Appl. Sci. 2021, 11, 12053.

[29] X. Zhou , X. Liu , D. Zhang , Z. Shen , W. Liu , J. Mater. Process. Technol. 2015, 222, 33.

[30] S. Liu , H. Guo , Materials 2020, 13, 3632. 32824450 10.3390/ma13163632 PMC7475986

[31] S. Jin , P. Shen , D. Zhou , Q. Jiang , Nanoscale Res. Lett. 2011, 6, 515. 21878133 10.1186/1556-276X-6-515 PMC3212054

[32] M. Pacella , P. W. Butler‐Smith , D. A. Axinte , M. W. Fay , Diamond Relat. Mater. 2015, 59, 62.

[33] Y. Hu , W. Cong , X. Wang , Y. Li , F. Ning , H. Wang , Composites, Part B 2018, 133, 91.

[34] J. D. R. Selvam , I. Dinaharan , R. S. Rai , In Encyclopedia of Materials: Composites, Elsevier, Amsterdam 2021, pp. 615‐639.

[35] A. Jain , R. Pankajavalli , S. Anthonysamy , K. Ananthasivan , R. Babu , V. Ganesan , G. S. Gupta , J. Alloys Compd. 2010, 491, 747.

[36] H. Aghajani , M. S. Motlagh , J. Mater. Sci.: Mater. Med. 2017, 28, 29. 28108957 10.1007/s10856-016-5843-x

[37] M. H. Nayfeh , In Fundamentals and Applications of Nano Silicon in Plasmonics and Fullerines, Elsevier, 2008, pp. 153‐167, 10.1016/C2016-0-00112-2.

[38] W. H. Bragg , W. L. Bragg , Proc. R. Soc. Lond. A 1913, A88, 428.

[39] S. Hussain , N. Tönnißen , E. S. Barreto , E. Gärtner , A. Kostka , H. Springer , V. Uhlenwinkel , N. Ellendt , Virtual Phys. Prototyping 2023, 18, 2269906.

[40] R. L. Batalha , V. E. Pinotti , O. O. S. Alnoaimy , W. C. Batalha , T. Gustmann , K. Kosiba , S. Pauly , C. Bolfarini , C. S. Kiminami , P. Gargarella , J. Mater. Res. 2022, 37, 259.

[41] V. Promakhov , A. Zhukov , M. Ziatdinov , I. Zhukov , N. Schulz , S. Kovalchuk , Y. Dubkova , R. Korsmik , O. Klimova‐Korsmik , G. Turichin , A. Perminov , Metals 2019, 9, 141.

[42] T. M. S. Ramachandran , R. G. Reddy , In: Situ Synthesis and Characterization of TiB_2_ and Ti‐Al‐B Composites , Supplemental Proceedings, TMS (Ed.) Wiley, Hoboken, 2014, 10.1002/9781118889879.ch8.

[43] S. Mollica , D. K. Sood , M. K. Ghantasala , R. Kothari , P. Evans , G. Collins , The 11th Australian Conference on Nuclear Techniques of Analysis and the 5th Vacuum Society of Australia Congress. Proceedings, RBS and XRD analysis of silicon doped titanium diboride films (Conference), 1999.

[44] T. L. Bergman , A. S. Lavine , F. P. Incropera , D. P. DeWitt , Fundamentals of Heat and Mass Transfer, 7th ed. John Wiley & Sons, Hoboken, NJ, 2011.

[45] D. Suzuki , F. Itoigawa , K. Kawata , T. Suganuma , T. Nakamura , Key Eng. Mater. 2012, 523–524, 131.

[46] T. Nakamoto , E. Shamoto , Y. Yamazaki , Y. Sirakata , Journal of JSME 2006, 72, 370.

[47] P. Weidmann , U. Weber , S. Schmauder , G. Pedrini , W. Osten , Meccanica 2016, 51, 279.

[48] V. L. Solozhenko , V. Z. Turkevich , W. B. Holzapfel , J. Phys. Chem. B 1999, 103, 2903.

[49] Z. Cao , J. Sun , K. Zhang , W. Ji , K. Cai , B. Li , B. Liu , C. Fan , Composites, Part A 2024, 185, 108318.

[50] V. A. Neronov , M. A. Korchagin , V. V. Aleksandrov , S. N. Gusenko , Journal of the Less Common Metals 1981, 82, 125.

[51] B.‐X. Dong , F. Qiu , Q. Li , S.‐L. Shu , H.‐Y. Yang , Q.‐C. Jiang , Nanomaterials 2019, 9, 1152. 31405228 10.3390/nano9081152 PMC6723659

[52] A. V. Getling , Bénard–Rayleigh Convection: Structures and Dynamics, World Scientific, Singapore 1998.

[53] D. Gu , Y. Shen , Mater. Des. 2009, 30, 2903.

[54] X. Lv , Z. Yin , Z. Yang , J. Chen , S. Zhang , S. Song , G. Yu , Recent Progress in Materials 2024, 6, 009.

[55] Element Six , Precision machining: giving toolmakers a competitive edge. PCD, PCBN, CVD diamond and single crystal diamond solutions, https://e6‐prd‐cdn‐01.azureedge.net/mediacontainer/medialibraries/element6/documents/brochures/element‐six‐metalworking‐brochure‐en.pdf?ext=.pdf, (accessed: October 2025).

[56] N. Christensen , Acta Chem. Scand. 1975, A29, 563.

[57] J. Kim , S. Kang , J. Alloys Compd. 2012, 528, 20.

[58] M. Pacella , D. A. Axinte , P. W. Butler‐Smith , M. Daine , Proc. CIRP 2014, 13, 387.

[59] M. Pacella , S. Silburn , A. Badiee , R. Najjari‐Saadatabadi , P. Ghosh , S. Chen , G. Matthews , J. Manuf. Processes 2024, 132, 891.

